# Coupled catalytic states and the role of metal coordination in Cas9

**DOI:** 10.1038/s41929-023-01031-1

**Published:** 2023-10-02

**Authors:** Anuska Das, Jay Rai, Mitchell O. Roth, Yuerong Shu, Megan L. Medina, Mackenzie R. Barakat, Hong Li

**Affiliations:** 1 Institute of Molecular Biophysics, Florida State University, Tallahassee, FL, USA.; 2 Department of Chemistry and Biochemistry, Florida State University, Tallahassee, FL, USA.; 3 Present address: Materials and Structural Analysis Division, Thermo Fisher Scientific, Hillsboro, OR, USA.; 4 These authors contributed equally: Anuska Das, Jay Rai, Mitchell O. Roth.

## Abstract

Controlling the activity of the CRISPR–Cas9 system is essential to its safe adoption for clinical and research applications. Although the conformational dynamics of Cas9 are known to control its enzymatic activity, details of how Cas9 influences the catalytic processes at both nuclease domains remain elusive. Here we report five cryo-electron microscopy structures of the active *Acidothermus cellulolyticus* Cas9 complex along the reaction path at 2.2–2.9 Å resolution. We observed that a large movement in one nuclease domain, triggered by the cognate DNA, results in noticeable changes in the active site of the other domain that is required for metal coordination and catalysis. Furthermore, the conformations synchronize the reaction intermediates, enabling coupled cutting of the two DNA strands. Consistent with the roles of conformations in organizing the active sites, adjustments to the metal-coordination residues lead to altered metal specificity of *A. cellulolyticus* Cas9 and commonly used *Streptococcus pyogenes* Cas9 in cells.

The use of clustered regularly interspaced short palindromic repeats (CRISPR) and associated protein 9 (Cas9) has revolutionized genome-engineering technology^1,2^. Efforts towards understanding the underlying molecular basis for its catalytic efficiency and specificity can greatly improve the utility of Cas9. The Cas9 endonuclease targets DNA that contains a region of 20–24 nucleotides (nt) (that is, a protospacer) complementary to the associated CRISPR RNA (crRNA) guide as well as a unique 3–8 nt segment (a protospacer adjacent motif (PAM)) downstream of the protospacer. A hallmark of Cas9 function is the DNA-triggered conformational changes that control the catalytic efficiency and substrate specificity^3^. Extensive structural and biophysical characterization has revealed that two conserved lobes, the recognition or nucleic-acid-binding (REC) lobe and the nuclease (NUC) lobe—each comprising roughly half of the protein—undergo concerted motions^3^. The REC lobe is primarily responsible for anchoring the repeat and anti-repeat helix between the crRNA and the trans-activating CRISPR RNA (tracrRNA) (or the tetraloop-linked helix in a single-guide RNA (sgRNA)) and the guide–DNA heteroduplex. Part of the REC lobe buttresses the guide–DNA heteroduplex as it enters the cleavage position and disengages after the cleavage. The NUC lobe includes two catalytic domains: RuvC and HNH, for cleaving the non-target strand (NTS) DNA and the target strand (TS) DNA, respectively. Concomitant with engagement of the REC lobe with the guide–DNA heteroduplex, the HNH domain undergoes a large conformational change to be docked onto the TS^4–7^. Mutations in the REC lobe, the RuvC–HNH linker or the guide–DNA heteroduplex that are known to change the catalytic efficiencies also impact the rate of HNH conformational placement^4,8–10^. Similar mutations impact the cleavage efficiency of both the TS and NTS to the same degree^4^. A correctly placed HNH domain, although not necessarily its catalytic activity, activates as well as allosterically controls the RuvC domain for cleavage of the NTS DNA^11,12^, suggesting a coordination between the HNH and RuvC domains. The structural basis for this coordination, however, remains elusive.

The HNH domain of Cas9 has a typical ββα-metal fold and contains the conserved Asn (on α) as well as Asp and His (on β1) catalytic residues^13^. The RuvC domain of Cas9 contains a six-stranded mixed β-sheet (541236, ↑↑↑↓↑↑) sandwiched by helices and hosts the conserved Asp (on β1), Glu (on β4) and His (on α_Α_) catalytic residues^13^. Divalent metal ions are required for catalysis mediated by both the HNH and the RuvC domain^14,15^, and, interestingly, for the conformational change of the HNH domain^6,8,16^. While a single divalent ion is required for HNH-mediated TS cleavage, two are required for RuvC-mediated NTS cleavage^13^. Metal ions, especially Mg^2+^, which supports optimal activities for both HNH and RuvC, are extremely sensitive to their coordination environment, which forms the chemical basis for many enzymes to confer substrate specificity^17^. Conformational changes in Cas9 can thus potentially perturb the metal-coordination environment, especially that of the two-metal RuvC centre, to influence the catalytic processes and vice versa. Although the catalytic metals in Cas9 have recently been placed at the expected sites in the HNH and the RuvC domains of Cas9 by structural biology methods^6,7^, how the metal-coordinated centres are influenced by conformational changes and how they evolve throughout the entire DNA-cleavage process remains unknown.

Current knowledge of Cas9 catalysis is limited to comparisons with homologous enzymes of single catalytic domains^13^ or computational simulations^16,18^. Although these studies provide valuable mechanistic insights into individual the catalytic centres they do not capture the potential coupling between the two. Do both catalytic centres reach similar catalytic stages in synchronization or separately? Although kinetic and mutational studies have suggested that conformational changes in the HNH domain trigger the activities of both nuclease domains, the structural basis for this coupling at the level of catalysis is not clear. The direct observation of structural changes in the intact active sites during the DNA-cleavage process can, therefore, reveal the chemical basis for the coordinated cleavage of both DNA strands.

The *Acidothermus cellulolyticus* Cas9 (AceCas9) enzyme belongs to the Type II-C subtype that is, generally, smaller in size but contains catalytic residues identical to those in the commonly used Type II-A Cas9 systems such as *Streptococcus pyogenes* Cas9 (SpyCas9). In addition, the RuvC domain used by AceCas9 is the sole catalytic domain of another commonly used type of CRISPR endonucleases, Cas12, such as Cpf1 (ref. 19). AceCas9 is moderately thermophilic and specific to DNA protospacers associated with a 5′-NNNCC-3′ PAM. Although the wild-type AceCas9 has slower rate of cleavage than that of SpyCas9, protein directed engineering has resulted in a catalytic enhanced AceCas9 that has a comparable cleavage rate to that of SpyCas9 (ref. 10). Interestingly, AceCas9 is sensitive to DNA methylation and does not cleave DNA with a 5′-NNN^m^CC-3′ PAM^20^. Whereas the unique and conserved biochemical properties of AceCas9 make it a potential candidate for biotechnology tool development, its inherently slower reaction rate also offers a chance for trapping catalytic intermediates.

Here we assembled the intact AceCas9 with a cognate DNA substrate (Fig. 1a,b) under reactive conditions and studied the structures of the AceCas9 complexes populated in two reaction mixtures via cryo-electron microscopy (cryo-EM). Using a technique analogous to time-resolved cryo-EM^21^, we captured five distinct states of active AceCas9 at 2.2–2.9 Å resolution, including two catalytic intermediates. The well-resolved density reveals the detailed coordination chemistry of the metal ions in the HNH and RuvC catalytic sites and how reactive states evolve throughout the DNA-cleavage process. The structural findings are complemented by consistent results from Cas9 activity assays in cells.

## Results

### Domain movements during catalysis

**Fig. 1 |:**
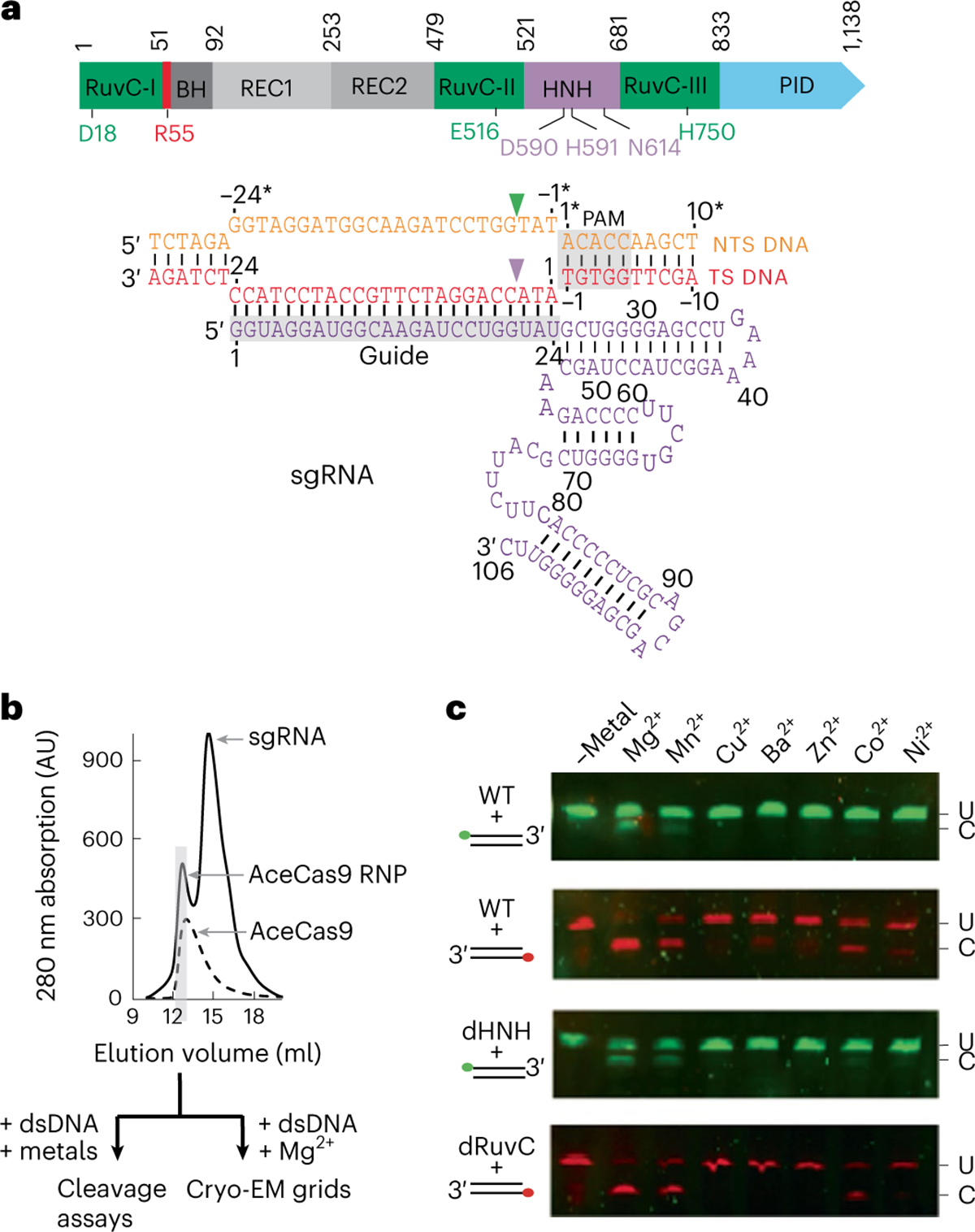
AceCas9 and its metal dependence. **a**, Top: domain organization of AceCas9 shown as coloured blocks in the direction from the N terminus to the C terminus. The regions corresponding to the structural domains are coloured and labelled, and the relevant residues are labelled. RuvC-I–RuvC-III, discontinuous segments of the RuvC domain; BH, bridge helix; REC1, nucleic acid-recognition domain 1; REC2, nucleic acid-recognition domain 2; HNH, HNH nuclease domain; PID, PAM interaction domain. Bottom: schematic diagram of the nucleic acids used in this study, shown as nucleotides in the predicted secondary structures. Cleavage sites for the NTS DNA by the RuvC domain and the TS DNA by the HNH domain are indicated by the green and purple downtriangles, respectively. The PAM and the guide region are highlighted in grey.The TS and NTS are numbered sequentially with NTS numbers denoted with asterisks. **b**, Overlay of the gel filtration profiles of the AceCas9 protein and its ribonucleoprotein (RNP) complex assembled with the sgRNA shown in **a**. Samples collected for biochemistry and cryo-EM analysis are highlighted by the grey shaded area. **c**, Cleavage results of double-stranded DNA (dsDNA) assembled with either TS DNA labelled with hexachlorofluorescein (HEX) (red) or the NTS oligonucleotide labelled with fluorescein amidites (FAM) (green) at 10 nM by AceCas9 or its catalytic mutants at 1 μM in the presence of various divalent ions at 10 mM. WT, wild-type AceCas9; U, uncleaved DNA substrate; C cleaved DNA substrate; dHNH, AceCas9 with deactivated HNH; dRuvC, AceCas9 with deactivated RuvC.

The recombinantly expressed full-length AceCas9, assembled with an in-vitro-transcribed sgRNA, was first tested for its dependence on metal ions using individually labelled DNA substrates (Fig. 1c). Whereas the cleavage of TS DNA by the HNH domain is supported by Mg^2+^, Mn^2+^ and Co^2+^, the cleavage of NTS DNA by the RuvC domain is supported by Mg^2+^ and Mn^2+^, suggesting that RuvC has a more stringent requirement for metal-coordination properties than HNH.

**Fig. 2 |:**
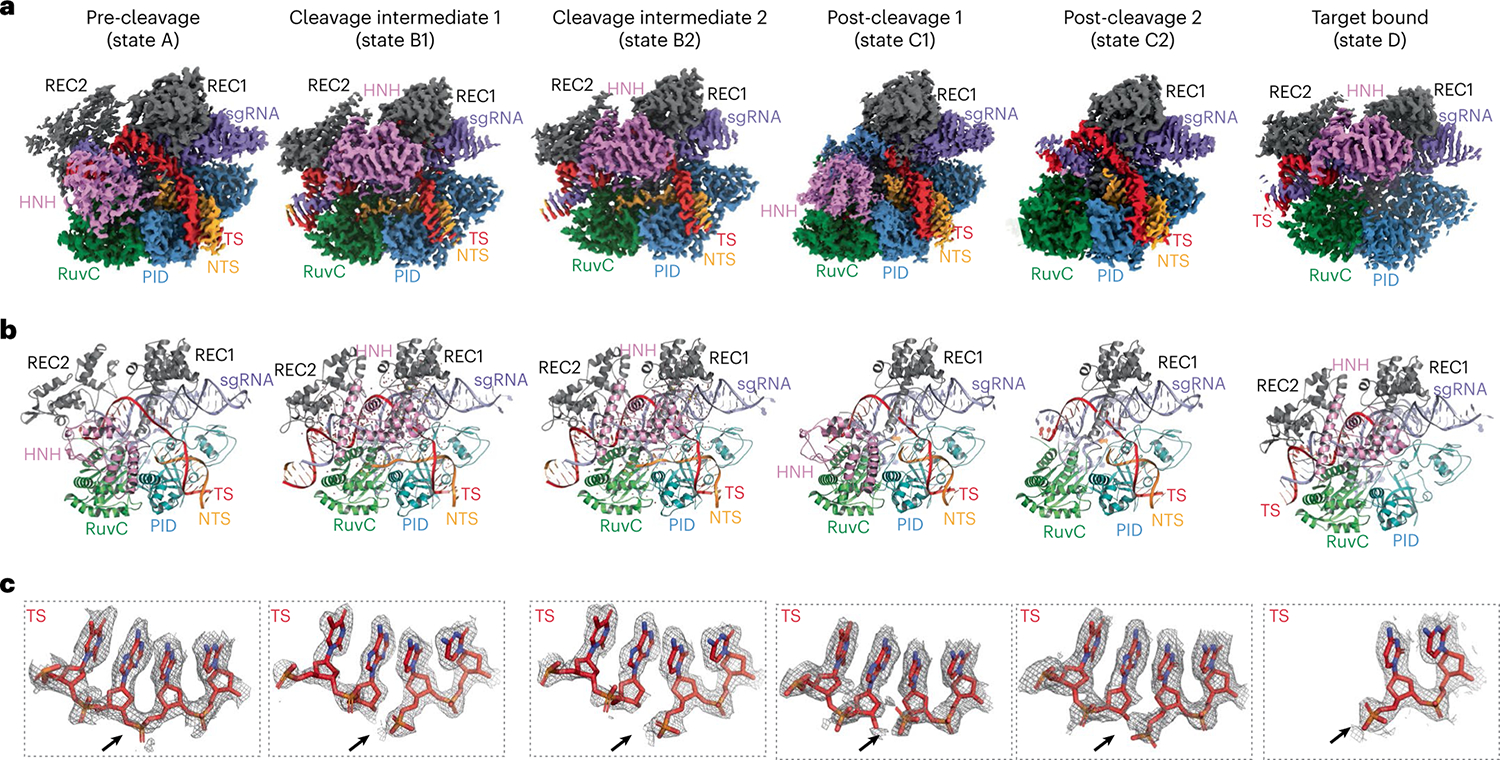
The observed cryo-EM structures of six reaction states. **a**, Electron potential density maps corresponding to the pre-cleavage (A), cleavage intermediates (B1 and B2), post-cleavage (C1 and C2) and target-bound (D) states. RuvC, RuvC nuclease domain. **b**, Cartoon representations of the structural models corresponding to the maps in **a**, where each domain or nucleic acid molecule is labelled in the same colour. **c**, Close-up views of the cleavage site of the TS DNA overlaid with the density for each of the corresponding states shown in **a** and **b**. The arrow indicates the position of the scissile phosphate bond.

To capture the structures of AceCas9 in reactive states, we assembled two active AceCas9–sgRNA–DNA complexes in a buffer containing Mg^2+^ that were allowed to react for different times (15–30 min) before preparation of the cryo-EM samples. Particles from the two independent data sets were sorted and classified extensively (Supplementary Figs. 1 and 2; Supplementary Table 1), which resulted in six different classes based on the location (or absence) of the HNH and REC2 domains. The excellent quality of density around the DNA cleavage sites in all the structures, especially that of the TS DNA, enabled the classes to be sorted into the following structures: pre-cleavage (state A), cleavage intermediate (states B1 and B2), post-cleavage (states C1 and C2) and target-bound (state D) (Fig. 2a–c).

**Fig. 3 |:**
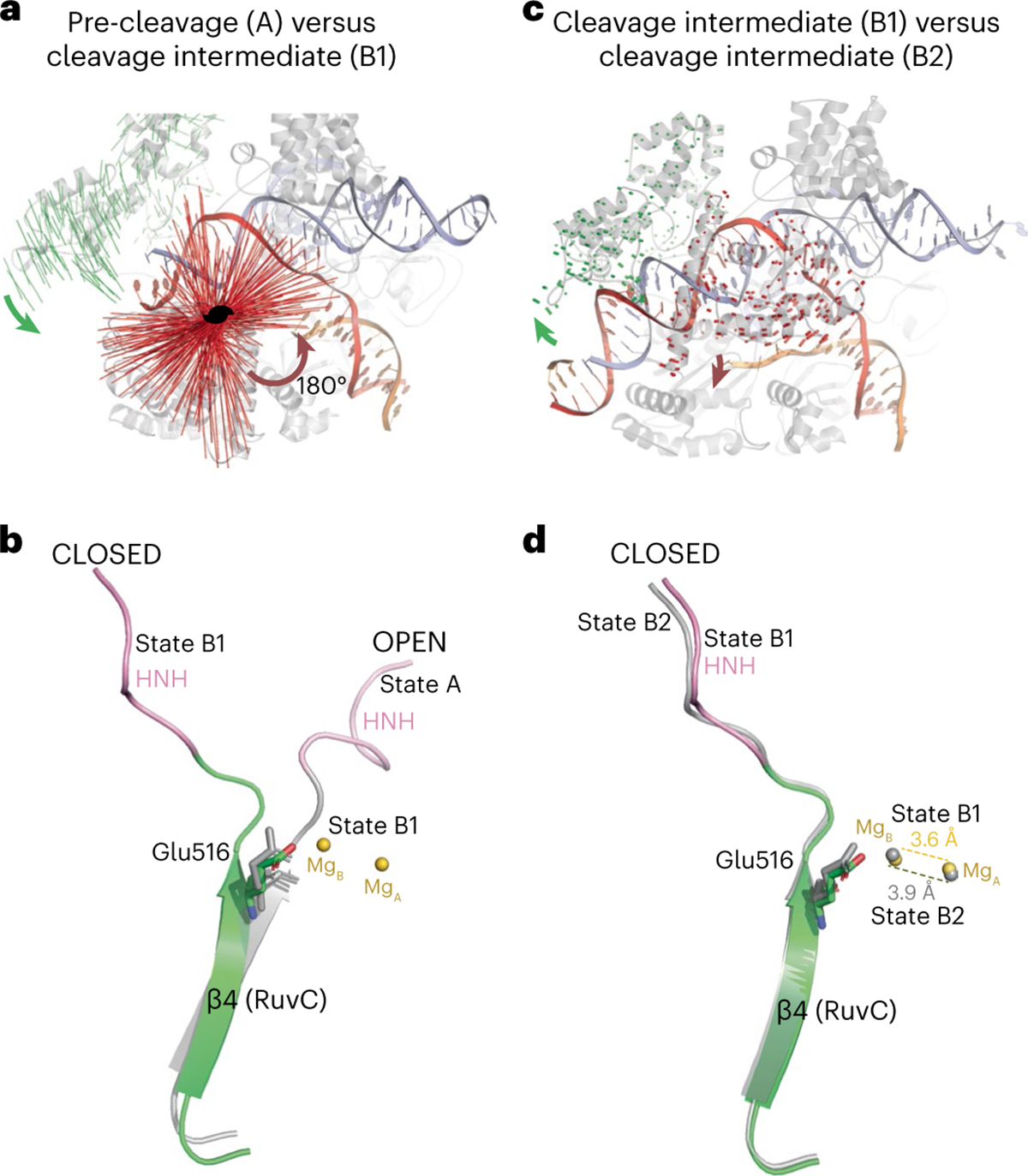
HNH conformation-linked changes in the RuvC active centre. Domain conformations are compared when the sgRNA between the two compared complexes is aligned. In **a**,**c**, for the two compared AceCas9 structures the red lines indicate the pair-wise displacement of Cα atoms within the HNH domain, and the green lines indicate the pair-wise displacement of Cα atoms within the REC2 domain. **a**, Comparison between the pre-cleavage structure (state A) and the catalytic intermediate structure (state B1). Curved arrows indicate the overall rotations of the HNH (red) and REC2 (green) domains from state A to state B1. The two-fold rotation symbol (in black) indicates the location of the axis of the two-fold rotation for the HNH domain. **b**, Comparison of β4 and Glu516 of the RuvC domain (shown as a stick model) between the pre-cleavage structure (state A, coloured in grey and pink) and the catalytic intermediate structure (state B1, coloured in green and pink). Metal ions A and B from intermediate state B1 are shown as gold spheres. **c**, Comparison between the two cleavage intermediate structures (states B1 and B2). Curved arrows indicate the overall rotations of the HNH (red) and REC2 (green) domains from state B1 to state B2. **d**, Comparison of β4 and Glu516 of the RuvC domain between the two catalytic intermediates (state B1, coloured in green and pink, and state B2, coloured in grey). Metal ions A and B from intermediate state B1 are shown as gold spheres, and those from B2 are shown as grey spheres. Dashed lines indicate the distance between the two metal ions in the two different states, as labelled.

The most notable structural difference among the various states is the location of the HNH and REC2 domains. Both the pre- and post-cleavage states (A, C1 and C2) (OPEN conformations) are characterized by HNH being either placed away from the target cleavage site or disordered. In these states, the REC2 domain is also loosely associated with the guide–target heteroduplex or is disordered. By contrast, the other three states (B1, B2 and D) (CLOSED confirmations) form a compact structure with both the HNH and REC2 domains closely engaging the DNA substrate (Fig. 2a–c). To reach the CLOSED conformation from the OPEN conformation, the HNH domain rotates 180° and the REC2 domain swings towards the RuvC domain (Fig. 3a). Surprisingly, the two reaction intermediate states (B1 and B2), although both are CLOSED and well ordered, exhibit subtle but clear domain differences (Fig. 3b). We tested the functional importance of the conformational transition from OPEN to CLOSED by mutating the residues that line the interface between the guide–target heteroduplex and the RuvC domain in bacterial cells (Supplementary Fig. 3). These mutations severely impacted the in vivo DNA-cleavage activities (Supplementary Fig. 3d), supporting the critical role of these regions in the conformational changes of the HNH and REC2 domains, and consequently, in their function.

HNH docking provides a final checkpoint for R-loop formation before catalysis. In the CLOSED conformation, a Type II-C Cas9-specific loop of ~12 amino acids immediately before the bridge helix advances towards the forked DNA to lock the R-loop for DNA cleavage (R-loop lock) (Supplementary Fig. 4). Residue Arg55 engages the last TS–NTS base pair, T(−1)–A(1*), through a cation–*π* interaction, resembling a tongue depressor, enabling the upstream NTS DNA nucleotides to feed into the RuvC active site. In the OPEN conformation, however, T(−1*) of the NTS returns to be stacked on A(1*) with Arg55 stacked on its base, also through a cation–*π* interaction (Supplementary Fig. 4), thereby directing the NTS away from the RuvC centre. The mutation of Arg55 to Trp or Tyr largely retained the activity, whereas that of Arg55 to Ala abolished the activity (Supplementary Fig. 4). The conserved presence of the R-loop lock, especially Arg55, in the Type II-C Cas9 may be a molecular strategy to compensate for their relatively weak DNA unwinding activity^22^.

### Dependence of metal coordination on domain movements

In the three OPEN states (A, C1 and C2), we did not observe convincing density that can account for the coordinated metal ions in either active site. This is in part due to the absence of the DNA substrate in the enzyme active sites that is known to play a critical role in coordinating metals^17^. However, as a result of HNH domain docking, we observed significant rearrangements of a metal-coordination ligand of the RuvC active centre that influence metal coordination.

**Fig. 4 |:**
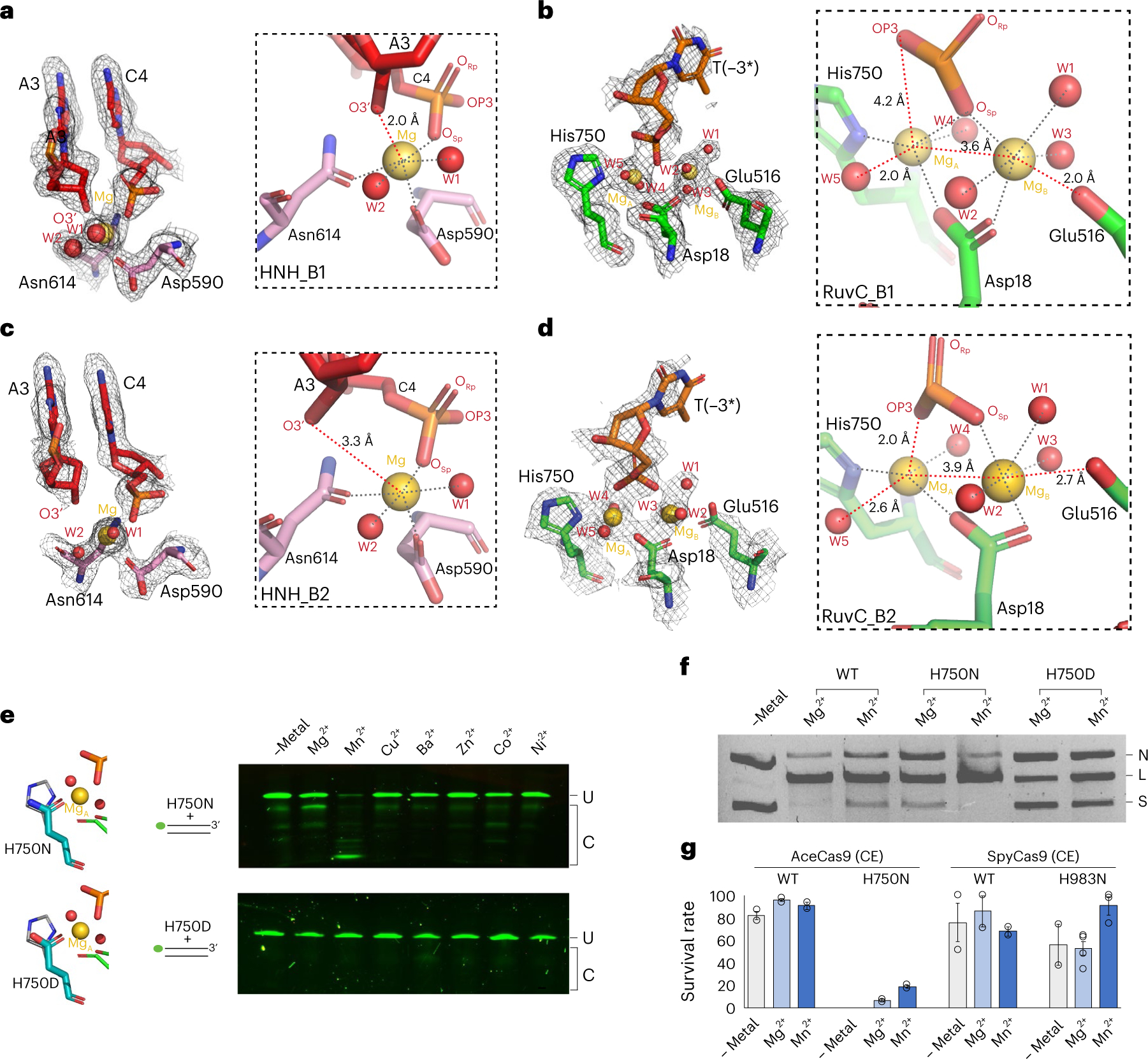
Structural changes of metal coordination and their impact on activity. **a**–**d**, Density and structures of the HNH catalytic centre of the intermediate B1 (**a**), the RuvC catalytic centre of the intermediate B1 (**b**), the HNH catalytic centre of the intermediate B2 (**c**) and the RuvC catalytic centre of the intermediate B2 (**d**). Density is shown via the grey mesh. Protein residues and DNA nucleotides are shown as coloured stick models; metal ions are shown as gold spheres; and water molecules are shown as red spheres. The insets in dashed boxes show close-up views of the metal-coordination environment, in which grey dotted lines indicate coordination distances of 1.7–2.2 Å and red dotted lines indicate the distances notably varied between the B1 and B2 intermediates. DNA residues are labelled by their names and corresponding numbers. **e**, In vitro DNA-cleavage activities on a dsDNA oligonucleotide substrate by the His750Asn (H750N) and His750Asp (H750D) variants in the presence of various divalent ions. The NTS DNA is labelled with the FAM fluorescence probe (green dot) at its 5′ end. The stick models represent putative structures of the mutated residues (teal) in comparison with wild-type His750 (grey). **f**, Comparison of DNA plasmid cleavage activities of AceCas9 (WT), H750N and H750D in the presence of Mg^2+^ or Mn^2+^ ions. RNP (500 nM) and DNA plasmid (6 nM) were used, and the reactions were incubated for 15 min at 50 °C. N, nicked; L, linearized; S, supercoiled plasmid DNA. **g**, Cell survival assay results of the H750N (for AceCas9) and H983N (for SpyCas9) variants in the presence of Mg^2+^ or Mn^2+^ ions. The Val709 to Ala mutation (V709A) of AceCas9 and the Lys918 to Asn mutation (K918N) of SpyCas9 are catalytically enhanced (CE) variants of the two Cas9 proteins and were used to assist stronger survival in non-optimal targeting (Supplementary Fig. 7). Raw images showing the formation of colonies are included in Supplementary Fig. 7. Survival rates are calculated from ratios of the colony-forming units on the arabinose–chloramphenicol plates to those of the chloramphenicol-only plates, as shown in Supplementary Fig. 3. Each experiment was performed in triplicate and the data are plotted with error bars that show the standard deviation. For AceCas9, *n* = 2 biologically independent trials were examined. For SpyCas9, *n* = 3 biologically independent trials were examined. Individual rates of survival are plotted as open circles, and the vertical bars show the mean ± standard deviation.

Glu516 of AceCas9 is a conserved coordination ligand to metal B of the RuvC centre (Figs. 3c,d and 4). It is located on the well-conserved β4 of the RuvC domain linked directly to the hinge of HNH, supporting its motion (Fig. 3c,d). In the OPEN conformation, irrespective of HNH placement, β4 of RuvC stays parallel with β1, causing the Glu516 side-chain to point away from metal B (Fig. 3c). In this conformation, RuvC remains inactive, even if DNA is placed in its active site. In the CLOSED conformation, however, Glu516 is repositioned to be perfectly aligned for chelating metal B (Fig. 3c). The conformation-mediated Glu516 repositioning is also evident, although to a lesser degree, between the two intermediate structures (Fig. 3d).

### Concerted HNH and RuvC reaction states

In state B1, HNH engages closely with the target DNA when the leaving 3′-hydroxyl remains coordinated with its metal (Fig. 4a). In the same state at the RuvC catalytic centre, Glu516 is poised to coordinate with metal B, which helps to bring the two metals close together at 3.6 Å (Fig. 4b). In state B2, however, where HNH is relaxed after cutting the target DNA (Fig. 4c), Glu516 retracts slightly, bringing metal B along with it, leading to a longer 3.9 Å metal-to-metal distance at the RuvC centre (Fig. 4d). The change in metal coordination in the RuvC catalytic centre as a result of the subtle rotation of HNH is consistent with the observation that HNH leads RuvC in cleavage^11,23^ and allosterically controls the RuvC activity^12^.

Examination of the metal-coordination geometries in simultaneously trapped intermediate structures further reveals coordinated reaction states between the HNH and the RuvC catalytic centres. In state B1, the single metal in the HNH centre has a near perfect octahedral coordination with six ligands: two water molecules, the carboxylate of Asp590, the carboxyamide of Asn614, the pro-S oxygen of the scissile phosphate (O_Sp_) and the leaving 3′-hydroxyl group (O3′) (Fig. 4a and Supplementary Fig. 5). Interestingly, to maintain coordination to the metal ion, the leaving nucleotide must adopt an unusual O4′-*exo* sugar pucker conformation that is uncommon in DNA (Supplementary Fig. 5). This active-site configuration is consistent with a mechanism where the metal ion both prepares the scissile bond for the nucleophilic attack and stabilizes the negatively charged pentavalent intermediate. The nearby His591 does not coordinate with the metal and acts as a general base to deprotonate the water nucleophile (Supplementary Fig. 5). This also is consistent with the fact that other octahedral-coordination metals can support the cleavage reaction (Fig. 1c). Strikingly, in state B2 when HNH undocks slightly (Fig. 3) and the distance between the leaving 3′-hydroxyl group and the metal increases from 2.0 Å in B1 to 3.3 Å in B2 if the nucleotide remains in a non-2′-*endo* sugar pucker conformation (Supplementary Fig. 5), leaving the metal with five ligands (Fig. 4c).

Structural changes in the water molecules surrounding those coordinated with the metal ion between the two intermediates are also observed (Supplementary Fig. 5). In particular, a water molecule bonded by both the pro-R oxygen of the scissile phosphate (O_Rp_) and a metal-coordinating water molecule in state B1 is moved nearly 4 Å away in B2 (Supplementary Fig. 5). Another water molecule bonded by Lys617 in state B1 interacts instead with the Gly618 carbonyl oxygen in B2 (Supplementary Fig. 5). Water rearrangement in the HNH active site may reflect the need for protonation of the leaving groups by the computationally predicted Lys617-equivalent residue of SpyCas9 (ref. 16). Consistent with the discontinuous density between the metal and the leaving 3′-hydroxyl group (Fig. 4c and Supplementary Fig. 5), the significant changes in reaction coordinates indicate that B2 follows B1 on the HNH reaction path.

The RuvC centre displays the same reaction progression as that of the HNH centre. It contains two well-coordinated Mg^2+^ ions that also display a different arrangement between the two reaction intermediates (Fig. 4b,d). In B1, metal A is coordinated with five ligands that include two water molecules, the carboxylate of Asp18, Nδ1 of His750 and the pro-S oxygen of the scissile phosphate in a trigonal bipyramidal geometry (Fig. 4b). Metal B engages six ligands that include three water molecules, the carboxylate groups of Asp18 and Glu516 as well as the pro-S oxygen in a perfect octahedral geometry (Fig. 4b and Supplementary Fig. 5). Notably, metal A is 3.6 Å from metal B, which is much shorter than that captured in substrate-bound complexes of two-metal enzymes^24^. The split coordination of the pro-S oxygen between the two metals is reminiscent of the reaction intermediate first proposed by Steitz and Steitz for two-metal enzymes^25^. In B2, where the HNH domain undocks slightly, the two metals move further apart to 3.9 Å and metal B loses its coordination with Glu516 (Fig. 4d and Supplementary Fig. 5). The change in the metal-to-metal distance is a hallmark of two-metal enzymes and is believed to be important for catalysis^17^. Interestingly, state B2 seems to capture a stable scissile phosphate oxygen–metal interaction where pro-S and nucleophilic oxygen coordinate with metals B and A, respectively (Fig. 4d and Supplementary Fig. 5). Although not previously observed, a computational simulation study has suggested that this structure is theoretically possible when the 3′-hydroxyl group is absent in the active site^18^. As with the HNH centre, the observed geometry and the well-connected density indicate that intermediate B1 precedes B2 on the RuvC reaction pathway, revealing a striking synchronization in reactive states between the two catalytic centres.

### Altered metal coordination impacts conformational changes

Given the described relationship between the active centres and AceCas9 conformations, we wondered if metal coordination can impact conformational changes. We mutated the metal-A-coordinating residue His750 to Asn (H750N) and Asp (H750D) and tested the metal-dependent activities of the variants. Unlike the wild-type AceCas9 which prefers Mg^2+^, the H750N variant prefers Mn^2+^, showing a good activity level and an enhanced 3′–5′ exonuclease activity (Fig. 4e,f and Supplementary Fig. 6a–d). A similar effect was also observed in RNase H^24^. Interestingly, although H750D mimics the active centre of the RuvC domain of Cas12 (all 13 subtypes except for V-K), it showed no preference for metals and demonstrated a significantly reduced DNA-cleavage activity with either Mg^2+^ or Mn^2+^ (Fig. 4e,f and Supplementary Fig. 6a–c). Furthermore, unlike several well-characterized Cas12 enzymes that show collateral DNase activities^26^, H750D did not cleave single-stranded DNA (ssDNA) (Supplementary Fig. 6c), supporting a tighter control of the RuvC active site in Cas9 than in Cas12.

We then used a previously developed cell survival assay that is known to depend on the conformational changes of Cas9 (refs. 10,27) to measure the activities of His750 variants in cells. Whereas H750N showed a near-wild-type activity in the presence of Mn^2+^ in vitro (Fig. 4e,f and Supplementary Fig. 6a–c), it had no growth in the cell survival assay (Supplementary Fig. 7a–d), indicating an impaired process of conformational changes. When the previously known Val709 to Ala mutation (V709A), which increases the rate of conformational changes^10^, was introduced into H750N (AceCas9–H750N/V709A), it restored the cell survival activity to a reasonable level, and interestingly, the Mn^2+^ condition outperformed the Mg^2+^ condition (Fig. 4g and Supplementary Fig. 7a–d), consistent with in vitro observations (Fig. 4e,f).

To investigate whether or not the observed relationship between the conformation and metal coordination in AceCas9 holds true in other Cas9 enzymes that have well-conserved active sites, we constructed the analogous H750N and V709A mutations, that is, H983N and K918N, in commonly used SpyCas9 (SpyCas9–H983N/K918N) and tested its metal-dependent activities in the same cell survival assay with an appropriate target (Supplementary Fig. 7). To our satisfaction, even though SpyCas9–K918N showed similar activity under both the Mg^2+^ and Mn^2+^ conditions (Supplementary Fig. 7b–d), which is probably due to the known superior catalytic efficiency of SpyCas9 (ref. 22), SpyCas9–H983N/K918N with Mn^2+^ significantly outperformed that of Mg^2+^ (Fig. 4g). We interpret this result as a strong support for the link between metal coordination and conformational changes.

## Discussion

**Fig. 5 |:**
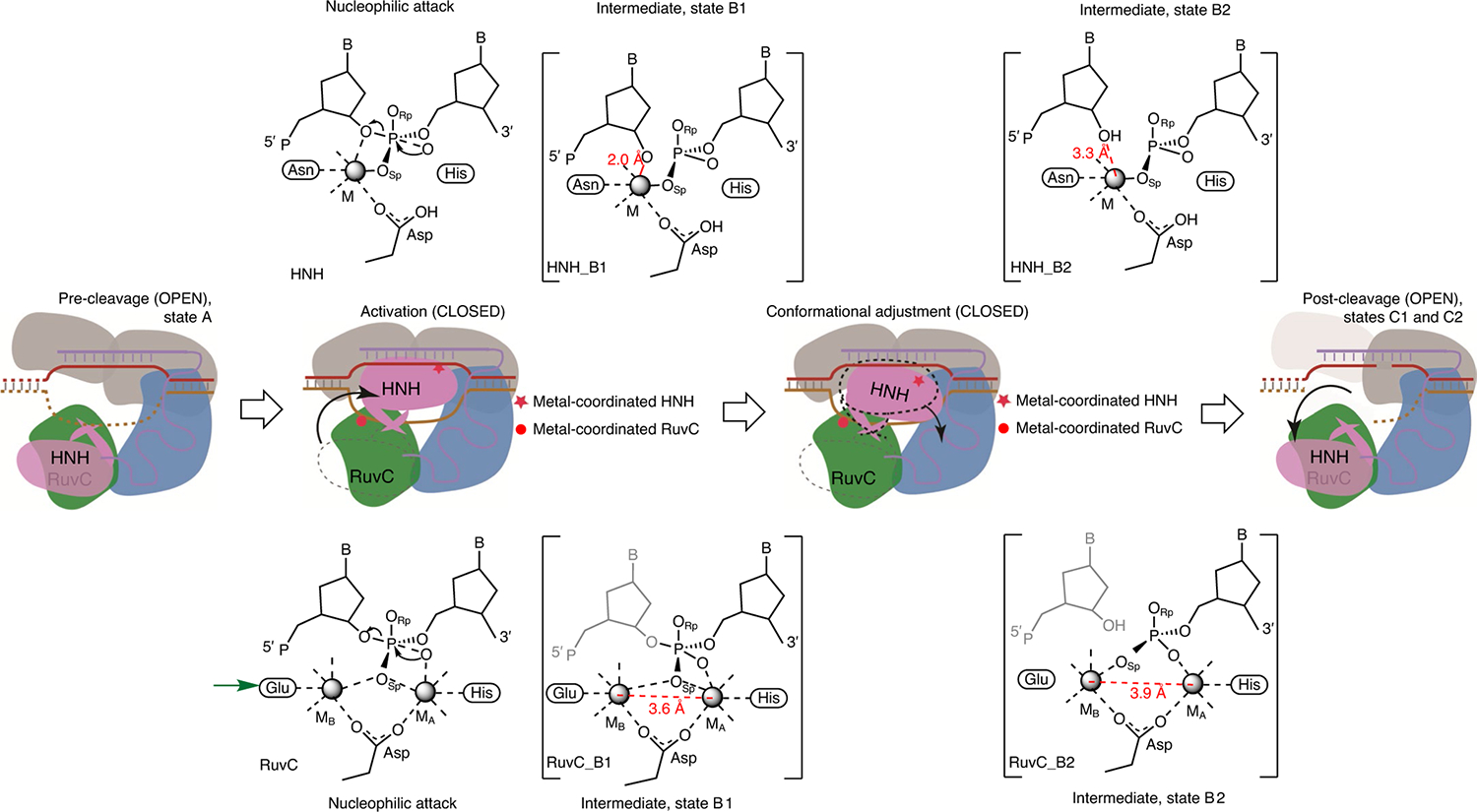
AceCas9 reaction scheme derived from the observed reactive states. From left to right, the correctly placed HNH domain from the OPEN (state A) to the CLOSED conformation initiates catalysis that begins by nucleophilic attack, followed by cleavage intermediates and, ultimately, relaxation back to the OPEN conformation (states C1 and C2). The same colouring scheme for domains and nucleic acids as that in Figs. 1 and 2 is used for the cartoon. The use of wild-type enzyme under reaction-prone conditions prevented capture of the substrate–enzyme complex, although two intermediates (states B1 and B2), as shown in square brackets, were trapped immediately after nucleophilic attack. ‘B’ and ‘P’ in nucleotide structures indicate base and phosphate, respectively. The grey-coloured leaving nucleotide at the RuvC site reflects the fact that it is not captured in the structures, possibly due to the known 3′–5′ exonuclease activity of RuvC or its disorder. A slight but noticeable rotation of the HNH domain from that in the first intermediate B1 to that in the second intermediate B2 (conformation adjustment) relaxes the HNH and RuvC metal-coordination spheres towards the enzyme–product state. The green arrow indicates the conformation-dependent glutamate of the RuvC centre. Red dashed lines with marked distances highlight key changes in the reactive states. M, metal; M_A_, metal A; M_B_, metal B.

The series of functional complex structures of AceCas9 at near-atomic resolutions reveal that both large and subtle conformation changes are necessary for catalysis (Fig. 5). We show that the catalytically relevant binding of metal ions to AceCas9 occurs only in the presence of the substrate as well as with the correct enzyme conformation. Two on-path reactive states were observed for the RuvC and HNH catalytic centres, respectively, in two cleavage intermediate states that differ slightly in conformation. Most remarkably, the reaction paths of the two catalytic centres are coupled to each other, revealing synchronized reaction progress (Fig. 5). The early intermediates for the HNH and RuvC centres are both characterized by high-resolution structural features that resemble the proposed catalytic intermediates for the one- and two-metal enzymes, respectively. The late intermediates, however, capture stable but clear features following DNA cleavage at both centres (Fig. 5). Our results provide a structural basis for understanding the previously hypothesized concerted DNA cleavage through HNH–RuvC communication^3^.

Several earlier studies of SpyCas9 clearly demonstrated that the active conformation of the HNH nuclease domain requires divalent ions that, interestingly, do not have to fully support catalysis^6,8,16^. Our results reveal a structural explanation. We showed that the active HNH conformation is necessary for the formation of the RuvC catalytic centre that, in turn, regulates the HNH conformation. Consistently, perturbations in the RuvC catalytic centre influence the rate of HNH activation, and, more significantly, reveal strategies for tuning Cas9 activities through metal coordination. In sequence-specific DNase such as restriction endonucleases, metal coordination is often sensitive to the cognate base pairs^17^. In Cas9 where sequence specificity is not required, however, metal coordination is exploited by enzyme conformational changes in control of its activity. As catalytic efficiency seems to be correlated with the canonical configuration of the metal ions, slight changes in the coordination environment, such as those between the two cleavage intermediates of AceCas9 or those due to guide–target mismatches, protein mutations or other compositional changes, can thus alter the activities through metal coordination.

In two-metal catalysed phosphoryl-transfer reactions, metal B is the perfect target for regulation since it is known to be more critical for catalysis than metal A^17^. The universally conserved glutamate (Glu516 in AceCas9) offers a long sidechain-tethered carboxylate and is exploited by Cas9 to regulate the RuvC activity. Subtle conformational changes in the HNH domain are observed to influence Glu516-mediated coordination to metal B. Metal A often has an imperfect coordination geometry and can be replaced by other catalytic groups such as an amino acid general base. Consistently, when the HNH site is super-imposed with the RuvC site, the HNH-associated Mg^2+^ more closely mimics metal B (Supplementary Fig. 8). Taking advantage of the better tolerance of perturbations by metal A, we are able to alter the metal specificity of Cas9 by mutating its coordinating histidine to asparagine. This variant outperforms the wild-type form in a Mn environment, supporting the impact of metal coordination on the rate-limiting step of Cas9 activity.

The catalytic residues in both catalytic centres are universally conserved in all Cas9 known so far, and they also bear a reasonable overall conservation (Supplementary Fig. 9). Studies of other Cas9 enzymes indeed support the observed coupling between the two active sites that we observed in AceCas9. Enzyme kinetics and Förster resonance energy transfer studies of SpyCas9 showed comparable rates of cleavage by HNH and RuvC, and, interestingly, that the HNH conformation allosterically controls the RuvC cleavage activity^11,12,23^. Note that mutations in the helix adjacent to residues that anchor the NTS DNA were found to impair the RuvC activity while HNH activity remains intact^12^, suggesting that the HNH conformational change exerts multiple levels of control over the RuvC active site. The two independently captured active SpyCas9 structures with metal ions bound, although at lower resolutions, show some resemblance to our observed intermediates B1 (ref. 6) and B2 (ref. 7), respectively, lending further support to the conserved coupling mechanism. Consistently, the analogous mutations in the RuvC centre of both AceCas9 and SpyCas9 led to the similar impact of the metals on the activity. The linked catalysis provides a means to ensure the coordinated cleavage of both DNA strands by two separate active sites.

Unlike Cas9, the RuvC domain in Cas12 (all 13 subtypes except for V-K) is required to cleave DNA or RNA (subtype V-I) and, interestingly, contains an aspartate instead of the catalytic histidine in the RuvC domain of Cas9. In addition, several well-characterized Cas12 enzymes, such as Cas12a, show strong ssDNA cutting following activation by cognate dsDNA, an activity that is apparently lacking in Cas9 (ref. 26). The only Cas12 enzyme that has been characterized for metal binding so far is Cas12i2, whose RuvC domain both processes guide RNA and cleaves DNA^28^. Crystallographic studies captured Cas12i2 in a pre-cleavage state with two metal ions bound, analogous^28^ to that in other two-metal enzyme–substrate complexes^29^, suggesting a similar catalytic mechanism. Although the AceCas9 His750Asp variant mimics the active centre and the metal dependency of Cas12i2, it shows a significantly reduced dsDNA activity and remains free of any ssDNA cleavage activity. The mechanism for the constitutively activated RuvC domain in the characterized Cas12 enzymes remains to be fully elucidated. However, the lack of an analogous HNH-domain-mediated control may explain the different RuvC activities between Cas9 and Cas12.

## Methods

### Protein and nucleic acid sample preparation

AceCas9 was cloned in the pET28b vector and expressed in the *Escherichia coli* BL21 strain, and was purified as described previously^20,30^. Briefly, the protein was purified via nickel affinity chromatography followed by heparin ion exchange. The heparin-purified protein was stored at −80 °C for further use. The wild-type and mutant proteins used for in vitro assays were purified further with size exclusion chromatography and stored at a 10 μM concentration. All of the mutations were introduced using a Q5 site-directed mutagenesis kit (New England Biolabs) with the primers listed (Supplementary Table 2).

The 94 nt sgRNA was transcribed in vitro with T7 RNA polymerase (Supplementary Table 2) and purified via phenol–chloroform extraction followed by gel filtration. The DNA used in cryo-EM and biochemical assays was purchased from Eurofins Genomics. The heparin-purified full-length protein was mixed with sgRNA at a 1:2 molar ratio, and the RNP was purified via size exclusion chromatography using a Superdex 200 increase column (Fig. 1b). The AceCas9–RNA–DNA ternary complex was reconstituted by adding pre-annealed dsDNA substrate DNA to the RNP at a 1:1 molar ratio. Magnesium chloride was made to a final concentration of 40 mM to the complex to initiate the nuclease activity.

### Cryo-EM sample preparation, data collection and 3D reconstruction

The reactive ternary complex was incubated at different temperatures for 15–30 min for an optimal grid-making condition. Two different reaction samples were used for cryo-EM studies. One was incubated at 50 °C for 30 min and the other at 37 °C for 25 min. Each sample, at approximately 2.0 absorption units at 260 nm (*A*_260_) and in a 4 μl volume, was applied to glow-discharged UltrAuFoil 300 mesh R1.2/1.3 grids (Quantifoil). The sample was allowed to absorb for 10 s on the grids followed by 2 s of blotting at 19 °C and 100% humidity. The grids were plunge frozen in liquid ethane cooled with liquid nitrogen using an FEI Vitrobot Mark IV system.

The two data sets were collected at the Laboratory for BioMolecular Structure of Brookhaven National Laboratory using a Krios G3i cryo transmission electron microscope equipped with a Gatan K3 direct electron detector (ThermoFisher Scientific). All images were collected at ×105,000 magnification with a corrected pixel size of 0.412 Å per pixel and a −1 to −2.2 μm defocus range in a counted super-resolution mode with a 20 eV energy filter. Motion correction was carried out in bin 2 using MotionCorr2 (ref. 31), and contrast transfer function (CTF) estimation was performed using the Gctf program^32^. The Laplacian-of-Gaussian-based auto-picking algorithm in RELION-4.0 (ref. 33) was used for particle picking. The cryoSPARC program^34^ was used for template-based particle picking and initial two-dimensional (2D) classification to eliminate bad particles. The gold-standard Fourier shell correlation at a value of 0.143 was used for resolution estimation. Local resolution was estimated using RELION-4.0 (ref. 33).

A total of 13,653 micrographs were collected from the two independent data sets and 12,757 were selected for further processing. Several rounds of 2D classification were executed to result in 5,817,494 good particles for three-dimensional (3D) classification using RELION-4.0 (ref. 33). The classes with similar features, especially with respect to the HNH domain and the REC2 domain, were combined and refined using customized 3D masks. Particles contributing to the class with HNH and REC2 domains CLOSED were further classified with 3D custom masks, leading to two distinct subclasses with improved resolution. Multiple rounds of CTF refinement were carried out to reach the final 3D reconstructions (Supplementary Figs. 1 and 2). Structural models were built using the Coot tools^35^ and refined using the Phenix software package^36^ to satisfactory stereochemistry and real-space map correlation parameters (Supplementary Table 1). Note that water molecules were only modelled based on both density and interaction chemistry in the two high-resolution structures.

### In vitro oligonucleotide cleavage assay

The wild-type or mutant AceCas9 was pre-incubated at 37 °C for 30 min with sgRNA at a 1:1 molar ratio to form the RNP. Annealing was carried out on both the FAM-labelled NTS DNA plus a molar excess of non-labelled TS as well as the HEX-labelled TS DNA plus a molar excess of non-labelled NTS by heating at 95 °C for 5 min before being cooled slowly to room temperature. The pre-annealed dsDNA substrate (10 nM) of the labelled strand was added to the RNP at a final concentration of between 500 nM and 1 μM along with divalent metal ions (10 mM). The reactions were incubated at 50 °C for a predetermined time before being quenched with the addition of an EDTA-containing stop buffer. The oligonucleotide products were resolved on an 8 M urea 15% polyacrylamide denaturing gel and visualized using a Bio-Rad ChemiDoc gel imaging system with 488 nm and 580 nm excitation wavelengths for the FAM- and HEX-labelled DNA, respectively.

### In vitro plasmid cleavage assay

The wild-type or mutant AceCas9 was mixed with sgRNA at a 1:1 ratio and incubated at 37 °C for 30 min to form the RNP. For the metal-dependent cleavage assay, different divalent metals at a concentration of 5–10 mM and the target plasmid at 6 nM were added to the RNP at a final concentration of between 500 nM and 1 μM before being incubated for different periods of time. The reactions were quenched by adding 5× stop buffer (25 mM Tris pH 7.5, 250 mM EDTA pH 8.0, 1% SDS, 0.05% w/v bromophenol blue, 30% glycerol). The products were resolved on 1% agarose gel and stained with ethidium bromide. For M13 ssDNA collateral cleavage assays, M13 ssDNA plasmid at a 2 μM concentration was added to the plasmid cleavage reaction and incubated for 50 min at 37 °C. The reactions were stopped and resolved on a 0.7% agarose gel and stained with ethidium bromide. The gels were visualized using the Bio-Rad ChemiDoc gel imaging system.

### Cellular survival assay

Cell survival assays were performed as previously described^27^ with modifications. For testing residues that are important for binding nucleic acids, the DNA sequence targeted in the p11-LacY-wtx1 encoding ccdB toxin is flanked by the minor PAM sequence 5′-NNNAC-3′ (p11-LacY-wtx1-AC) for AceCas9. The AceCas9-encoding plasmid contains the previously characterized Glu839Arg and Glu840Tyr mutation (RY)^37^ (AceCas9-RY) that enables survival in the cell containing the minor 5′-NNNAC-3′ PAM and a graded survival activity (Supplementary Fig. 3a–d). Additional AceCas9 mutations were constructed on the RY background. For testing metal-dependent activities, the DNA sequence targeted in the p11-LacY-wtx1 encoding ccdB toxin is flanked by the major PAM sequence 5′-NNNCC-3′ (p11-LacY-wtx1-CC) for AceCas9. This plasmid can also be targeted by SpyCas9 using the nearby 5′-NGG-3′ PAM sequence for SpyCas9. To perform the survival assay, *E. coli* BW25141 (a gift from D. Edgell) containing p11-LacY-wtx1-AC or p11-LacY-wtx1-CC was transformed with plasmids (100–200 ng) encoding the Cas9 or variants. The cells were recovered in super optimal broth at 37 °C for 30 min, followed by induction with isopropyl β-D-1-thiogalactopyranoside (0.05 mM) and recovered for additional 1 h. For testing the requirement for different metal ions, cells transformed with plasmids were grown in SOB media (0.5% (w/v) yeast extract, 2% (w/v) tryptone, 10 mM NaCl, 2.5 mM KCl) substituted with no or different metal ions (supplied at a final concentration of 5 mM) for 30 min with shaking at 37 °C before isopropyl β-D-1-thiogalactopyranoside (0.05 mM) was added, and recovery continued for an additional 1 h. The cells were then plated in chloramphenicol (52 μg ml^−1^) or on plates containing chloramphenicol–arabinose (10 mM). The plates were incubated at 37 °C for 16–20 h and then the colony-forming units were counted on each plate. Survival percentages were calculated as the ratio of colony-forming units on the chloramphenicol–arabinose plates to that of the chloramphenicol-only plates. The percentages with errors from three technical replicates were plotted using Microsoft excel.

### Reporting summary

Further information on research design is available in the Nature Portfolio Reporting Summary linked to this article.

## Supplementary Material

Supplementary Materials

## Data Availability

The atomic coordinates and associated density maps have been deposited at the Protein Data Bank with accession codes 8D2N (pre-cleavage), 8D2L (cleavage intermediate 1), 8D2K (cleavage intermediate 2), 8D2Q (post-cleavage 1), 8D2O (post-cleavage 2) and 8D2P (target-bound), and at the Electron Microscopy Data Bank with the accession codes EMD-27143 (pre-cleavage), EMD-27142 (cleavage intermediate 1), EMD-27141 (cleavage intermediate 2), EMD-27146 (post-cleavage 1), EMD-27144 (post-cleavage 2) and EMD-27145 (target-bound). All other data are available from the authors upon reasonable request. Source data are provided with this paper.
