# Supplementary material for: Coupled catalytic states and the role of metal coordination in Cas9
Source: Nat Catal. Author manuscript; Available in PMC 2024 Feb 12. (PMC10861241; doi:10.1038/s41929-023-01031-1)
Supplement: Supplementary Materials [file NIHMS1960711-supplement-Supplementary_Materials.pdf]

Table of Contents

Supplementary Figure 1. Workflow of single particle reconstruction of the active AceCas9 ternary complexes.

Supplementary Figure 2. Map assessment of the cryoEM reconstructions.

Supplementary Figure 3. Cell survival assays for roles of residues engaging the guide RNA:protospacer heteroduplex.

Supplementary Figure 4. R-loop lock structure in different functional states and functional assay results.

Supplementary Figure 5. Modeling coordination geometry at the HNH and RuvC centers.

Supplementary Figure 7. Metal-dependent activities in bacterial cells.

Supplementary Figure 8. Comparison of active site geometry between HNH and TraI relaxase (PDB ID: 2A0I) and between HNH and RuvC.

Supplementary Figure 9. Comparison of sequences of the RuvC and HNH domains for the known Cas9s.

Supplementary Table 1a. Statistics of cryo-EM data collection processing

Supplementary Table 1b. Statistics of model refinement and data deposition

Supplementary Table 2. DNA or RNA Oligos used for this study

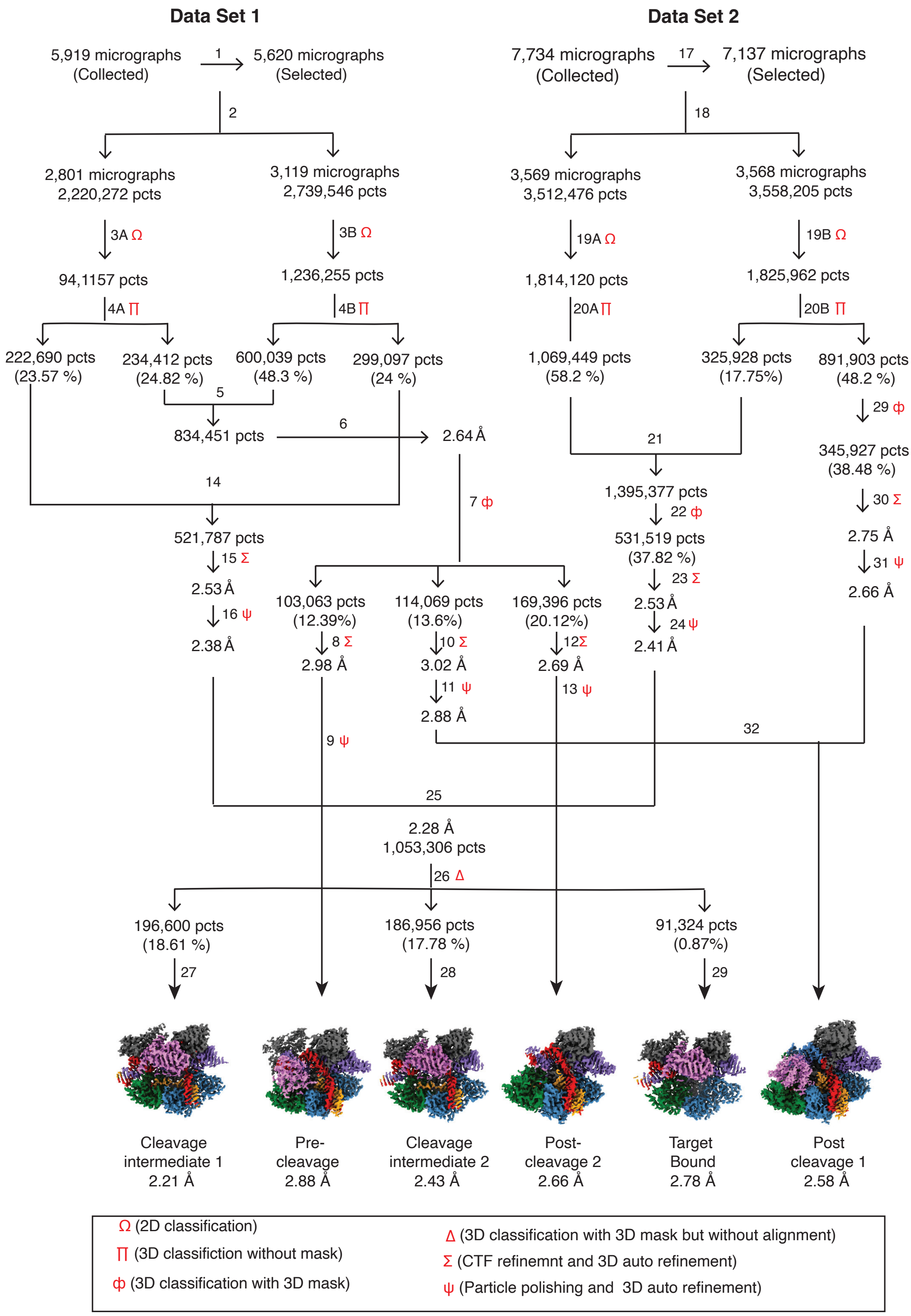

Supplementary Figure 1

Supplementary Figure 1. Workflow of single particle reconstruction of the active AceCas9 ternary complexes. Symbols in red represent the type of processes outlined in the textbox below. Numbers next to arrows account the steps of reconstruction and the percentage indicate those of particles to the steps above.

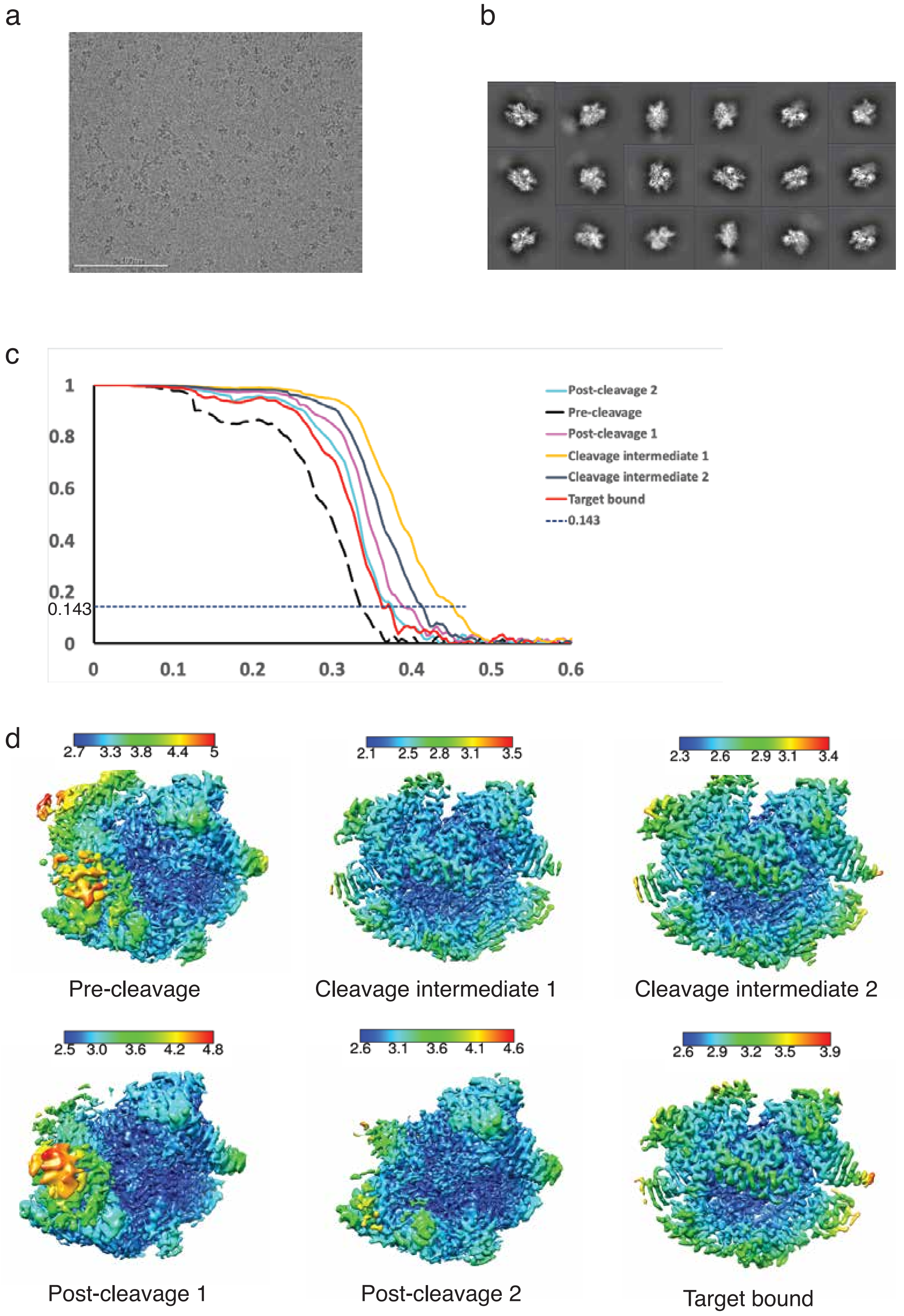

Supplementary Figure 2

Supplementary Figure 2. Map assessment of the cryoEM reconstructions. (a) Representative micrograph of the active AceCas9 ternary complex. Scale bar indicates 100 nm. (b) Select 2D class averages obtained from all particles used in reconstruction. (c) Fourier Shell Correlation (FSC) curves of the final classes that are colored and labeled, respectively. (d) Local resolution of the final six refined maps. The color bars define the resolution range.

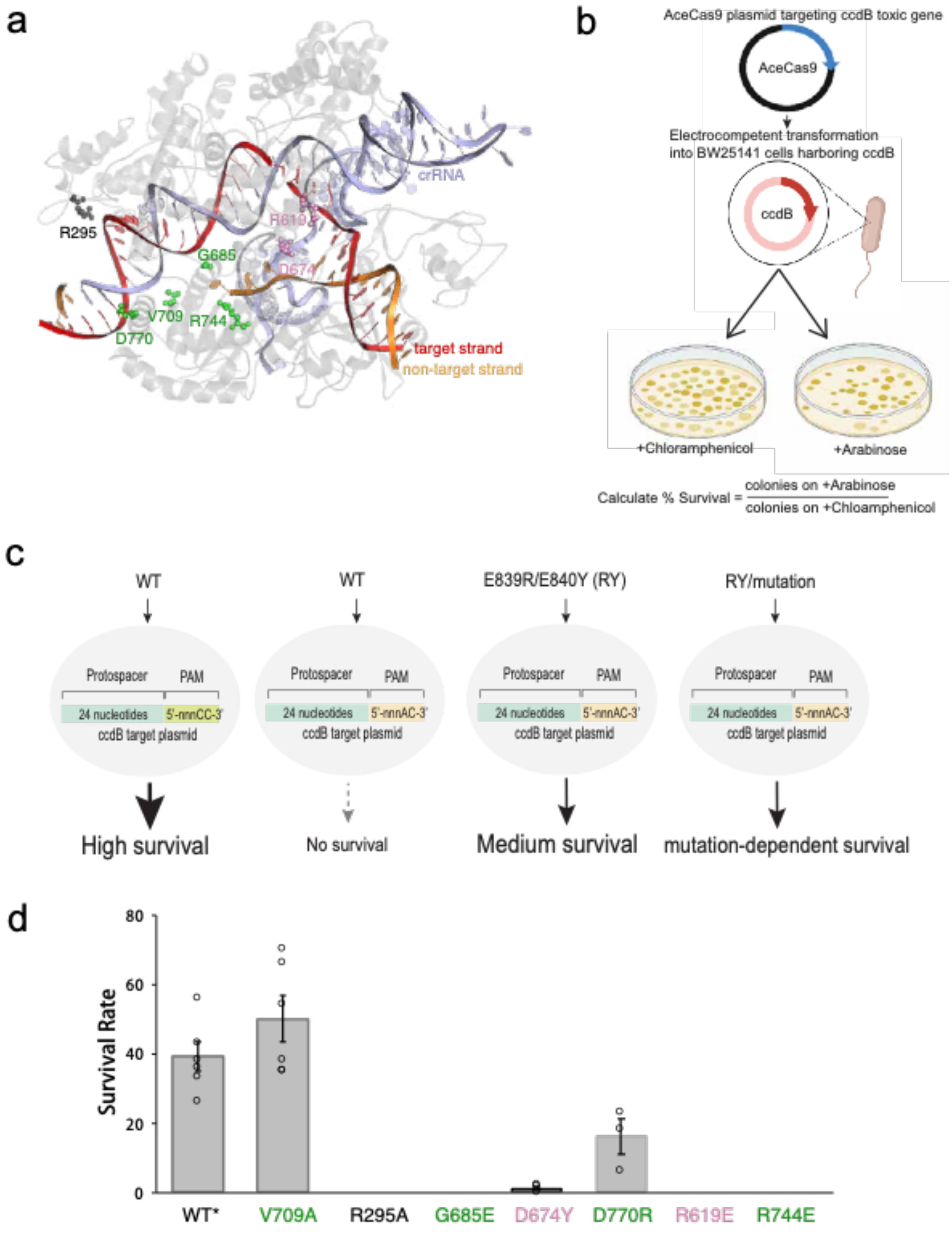

Supplementary Figure 3

Supplementary Figure 3. Cell survival assays for roles of residues engaging the guide RNA-spacer heteroduplex. (a) Locations of the residues in the AceCas9 ternary complex shown in colored spheres and labeled. The same color scheme as in Figures 1 and 2 is used. (b) Schematic of the enzymatic activity measurement in bacteria by a cell survival assay (Materials and Methods). Created with biorender.com (c) Rationales for using the AceCas9 E839R/E840Y variant to perform cell survival assays in cells harboring the DNA target with the minor (5′-NNNAC-3′) PAM. (d) Percent of survival for the indicated mutations on the AceCas9 E839R/E840Y variant versus that of the E839R/E840Y variant (labeled as WT*). n=4 biologically independent experiments for WT*, n=3 for V709A and D770R, n=2 for R295A, G685E, D674Y, R619E and R744E were examined. Individual rates of survival are plotted as open circles and the means as vertical bars +/- standard deviations.

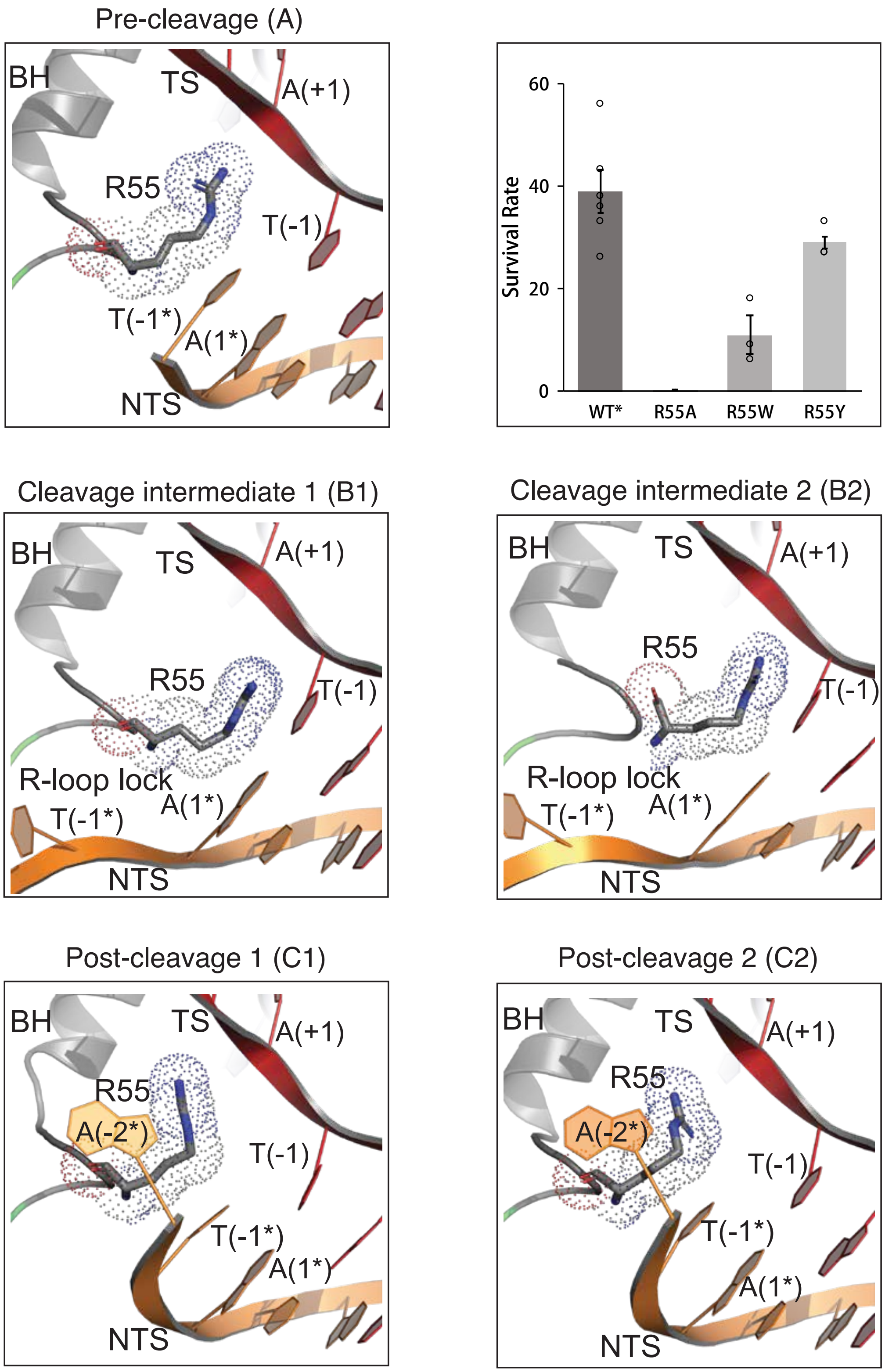

Supplementary Figure 4

Supplementary Figure 4. R-loop lock structure in different functional states and functional assay results. The R-loop lock residue Arg55 (R55) is shown in stick model with dotted van der Waals surface at the fork formed between the nontarget strand (NTS) and the target strand (TS) DNA in R-loop. Each functional state is labeled as in Figure 2. The same scheme as shown in Supplementary Figure 3a-c by using the AceCas9 E839R/E840Y variant to perform cell survival assays in cells harboring the DNA target with the minor (5′-NNNAC-3′) PAM is used. The calculated percent of survival for the indicated mutations on the AceCas9 E839R/E840Y variant (R55A, R55W, R55Y) versus that of the E839R/E840Y variant (labeled as WT*) are shown in bar diagram. n=4 biologically independent experiments for WT*, n=3 for R55W, n=2 for R55A, & R55Y were examined. Individual rates of survival are plotted as open circles and the means as vertical bars +/- standard deviations.

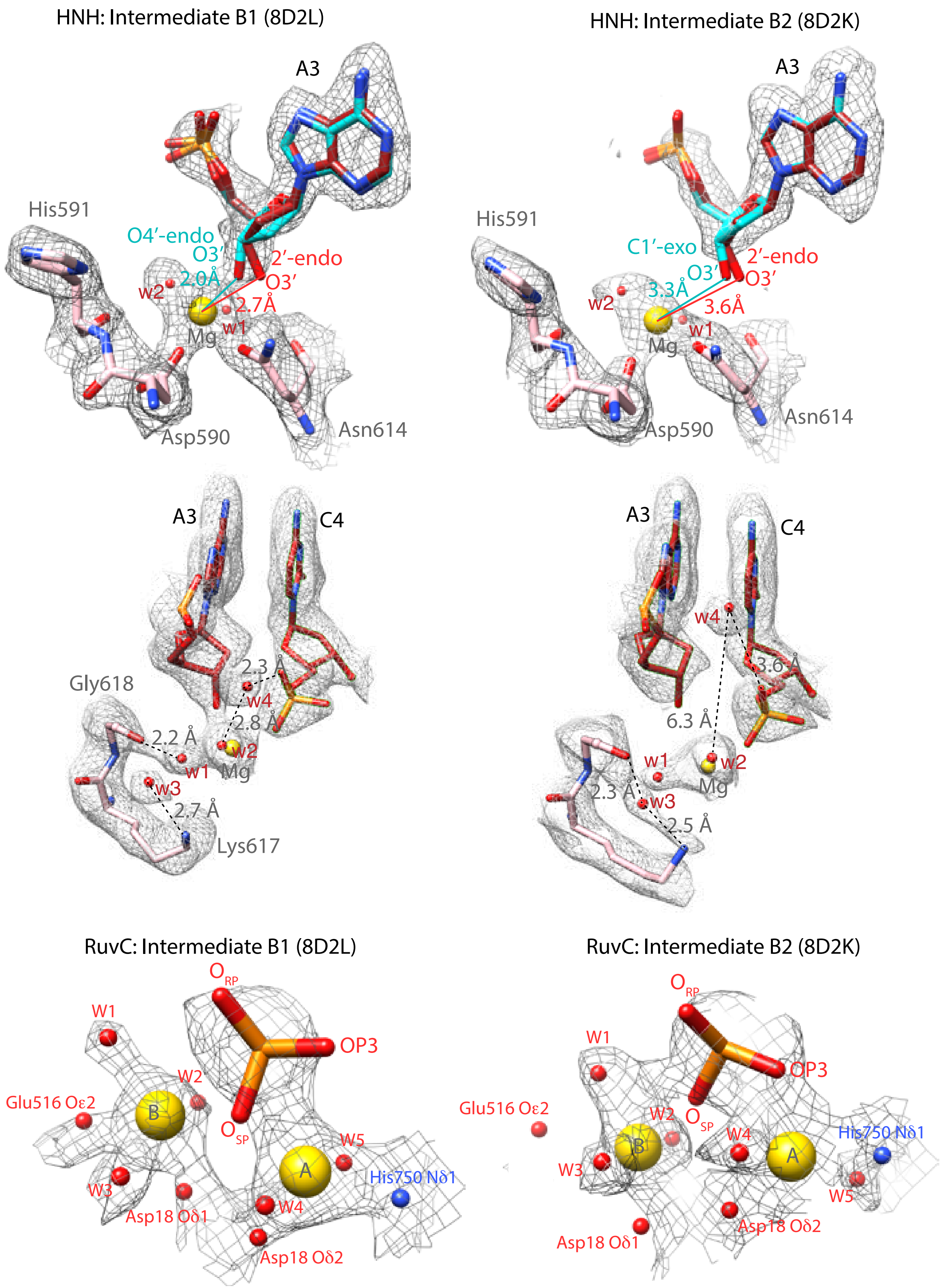

Supplementary Figure 5

Supplementary Figure 5. Modeling coordination geometry at the HNH and RuvC centers. Water molecules are denoted as W# with arbitrarily assigned numbers. Upper, close-up views of the HNH cleavage site including the leaving nucleotide of the 5' product overlaid with density for intermediate state B1 (left) and B2 (right), respectively. Correctly modeled and refined leaving nucleotide, A3, is colored in cyan whereas the same nucleotide in 2'-endo sugar pucker conformation commonly found in DNA nucleotides is shown in red. The refined sugar pucker conformations that fit density well are labeled in cyan next to the nucleotide. The distance between the 3'-hydroxyl group to the metal ion is labeled for both states in cyan for the refined conformation and in red for the 2'-endo sugar pucker model, respectively. Middle, movement of water molecules surrounding the metal coordination center from intermediate B1 (left) to intermediate B2 (right). Dashed lines indicate contacts that vary between the two intermediates. Lower, amplified views of the RuvC cleavage site with metal ions and coordination ligand atoms overlaid with density. Note the positions of atoms associated with protein residues are also constraint by sidechain conformations.

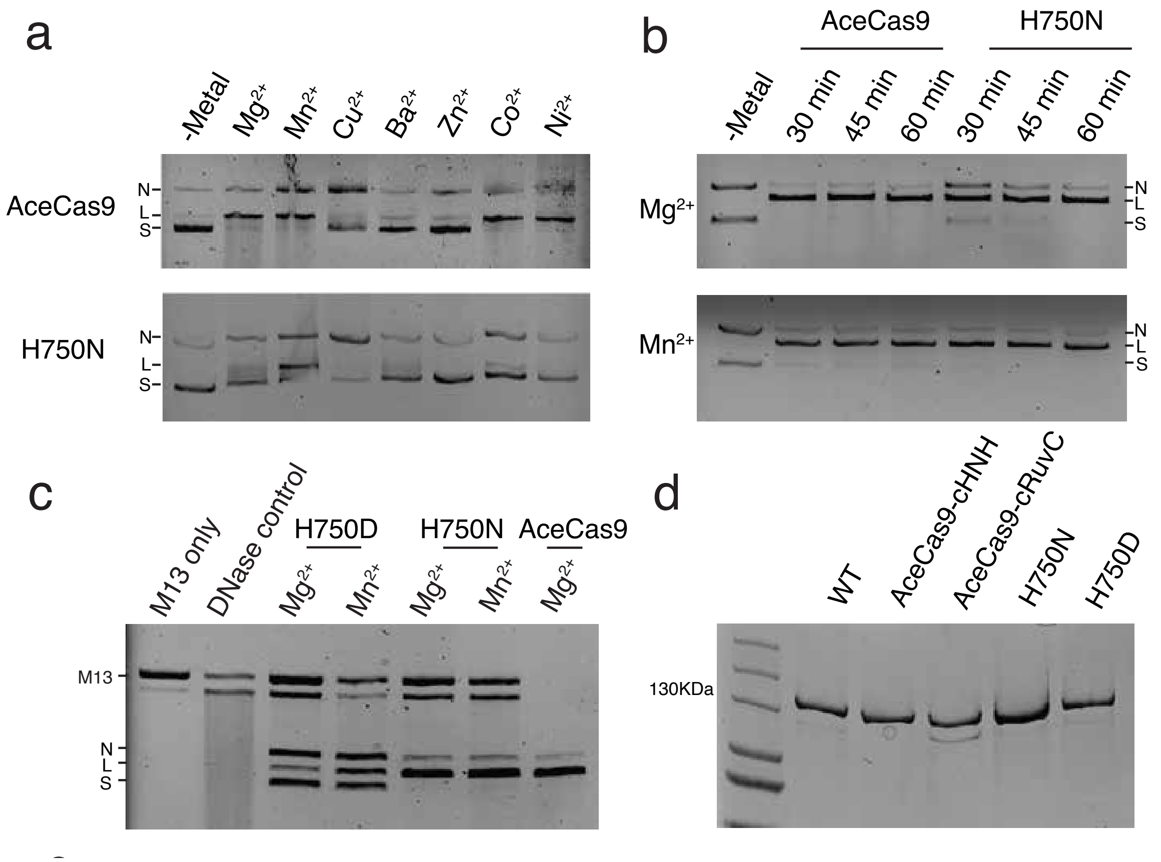

Supplementary Figure 6. Plasmid cleavage assay with AceCas9 wild type and mutants. (a) Target plasmid cleavage activities of AceCas9 and His750N with different divalent metals. "N" denotes nicked, "L" denotes linearized, and "S" denotes supercoiled plasmid DNA. (b) Comparison of DNA plasmid cleavage activities of AceCas9 and H750N with Mg^{2+} and Mn^{2+} at different timepoint. (c) M13 ssDNA cleavage (collateral activity) of H750D and H750N in presence of Mg^{2+} or Mn^{2+} and a cognate plasmid substrate. Turbo DNase cleavage of M13 is used as the DNase positive control. No M13 ssDNA was added to the reaction with the wild-type AceCas9. (d) Coomassie Blue stained SDS-PAGE of the proteins used in this study. The experiments (a)-(d) were repeated two times with similar results. Uncropped scans of gels are included as Supplementary Data file 1.

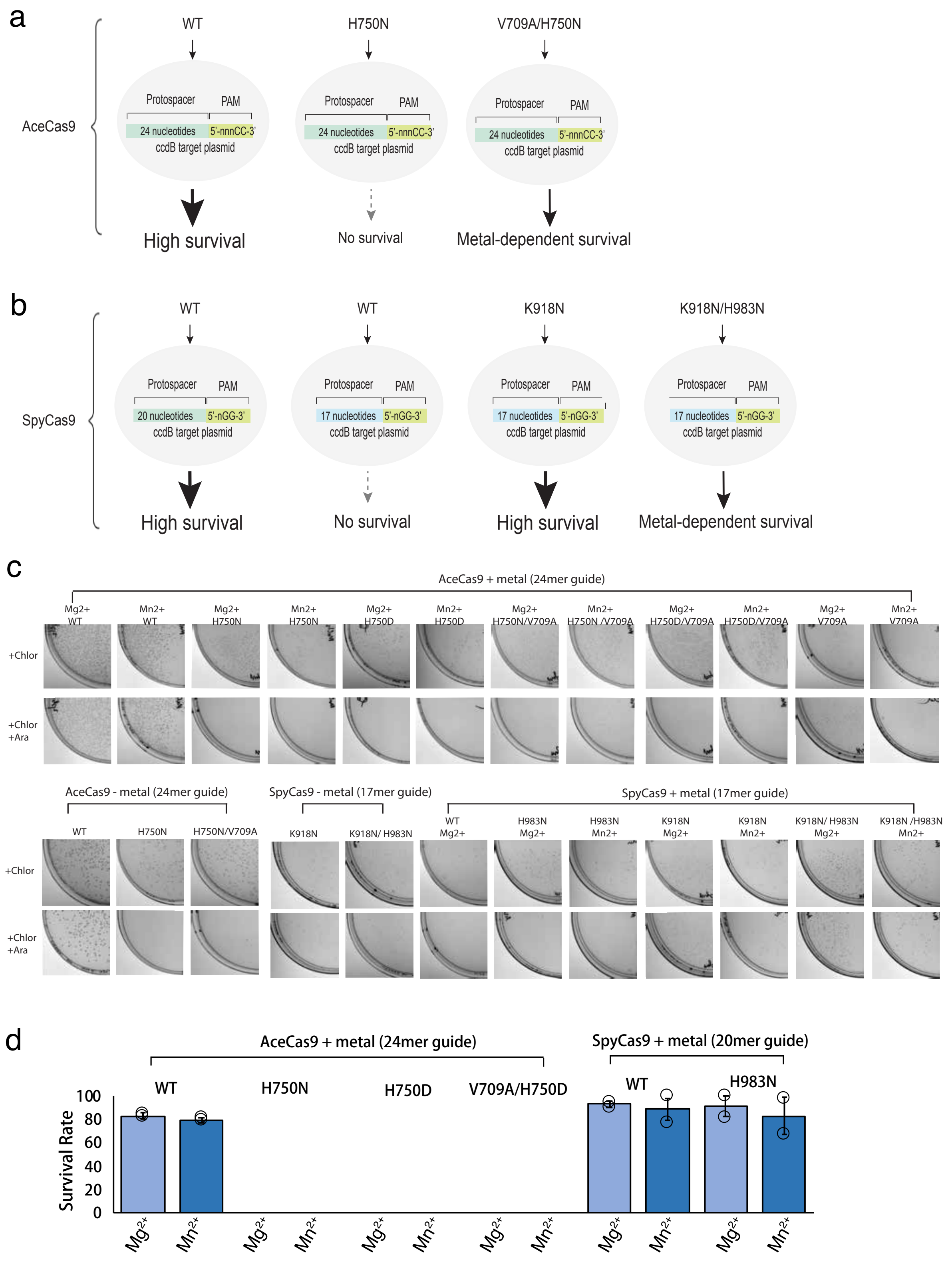

Supplementary Figure 7

Supplementary Figure 7. Metal-dependent activities in bacterial cells. (a). Rationales to perform metal dependent cell survival assay with the catalytically enhanced (CE) AceCas9 (V709A). (b) Rationales to perform metal dependent cell survival assay with the catalytically enhanced (CE) SpyCas9 (K918N). (c) Example plates obtained in bacterial survival assay with AceCas9 and SpyCas9 and their mutants in the presence and absence of metal ions. Either no or 5 mM metal ions ($MgCl_2$ or $MnCl_2$) was added to the recovery media for cells transformed with plasmids expressing wild-type (WT), V709A, H750N, H750D, V709A/H750N or V709A/H750D of AceCas9 or wild-type (WT), K918N, or K918N/H983N of SpyCas9. Agar plates containing chloramphenicol alone are labeled +Chlor and those containing both chloramphenicol and arabinose are labeled +Chlor, +Ara. Colony forming units were counted and used to calculate the rate of survival. (d). Calculated survival rates in different metals for the indicated wild-type or mutants of the two Cas9s. n=2 biologically independent experiments were examined. Individual rates of survival are plotted as open circles and the means as vertical bars +/- standard deviations.

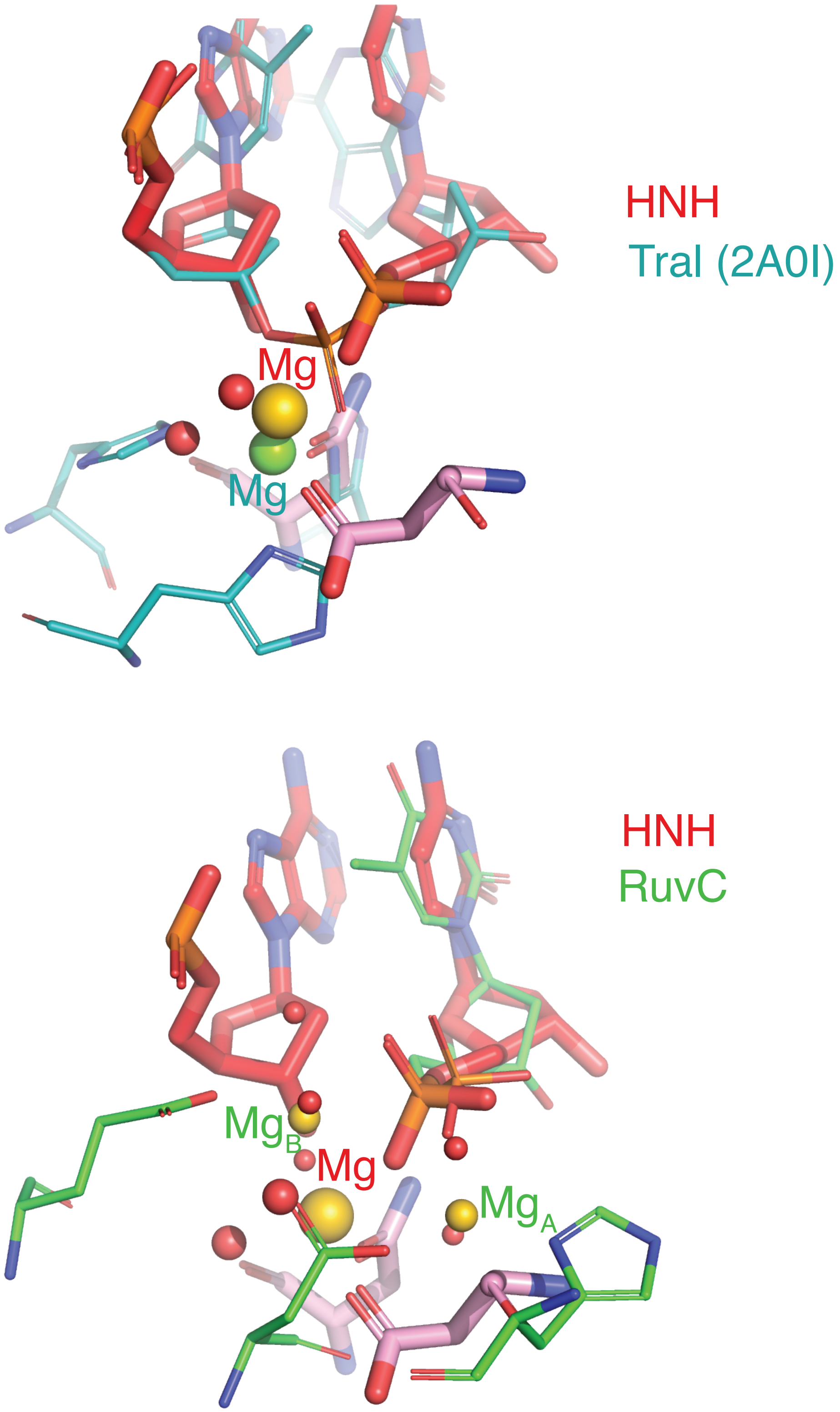

Supplementary Figure 8

Supplementary Figure 8. Comparison of active site geometry between HNH and TraI relaxase (PDB ID: 2A0I) and between HNH and RuvC. Residues of TraI are shown as think teal sticks and the bound metal is drawn as a green sphere. Residues of HNH are shown as thick red sticks and the bound metal is drawn as a gold sphere. Residues of RuvC are shown as thin green sticks, metals are shown as small gold spheres, and water are shown as small red spheres.

Pair-wise amino acid sequence identity of the known Cas9 (%) for RuvC/HNH

	AceCas9	AnaCas9	Nme1Cas9	SpyCas9	SauCas9	CdiCas9	CjeCas9
**AceCas9**	100.0	22.3/27.9	16.8/16.7	13.2/12.5	14.5/15.9	22.7	15.9
**AnaCas9**		100.0	14.8/17.5	15.6/11.8	12.6/12.6	36.0	13.9
**Nme1Cas9**			100.0	15.3/24.6	26.1/33.3	18.6	24.8
**SpyCas9**				100.0	16.8/21.9	14.6	16.4
**SauCas9**					100.0	14.9	21.0
**CdiCas9**						100.0	14.7
**CjeCas9**							100.0

HNH domain

```
Nme1Cas9HNH     ----------------------------------YFPNFVGEPKSKDILKLRLYEQQHGK
SauCas9HNH      -------------------------------------TTGKENAKYLIEKIKLHDMQEGK
SpyCas9HNH      IEMARENQTTQKGQKNSRERMKRIEEGIKELGSQILKEHPVENTQLQNEKLYLYYLQNGR
AceCas9HNH      ------ASESRERQAEEEAARRAHRKANDRIRAELRASGLSDPSPADLVRARLLELYDCH
AnaCas9HNH      -------SERMADERDKANRRRYNDNQEAMKKIQRDYGKEGYISRGDIVRLDALELQGCA
                                                                 :

Nme1Cas9HNH     CLYSGKEINLG-RLNEKGYVEIDHALPFSRTWDD-SFNNKVLVLGSENQNKGNQTPYEYF
SauCas9HNH      CLYSLEAIPLEDLLNNPFNYEVDHIIPRSVSFDN-SFNNKVLVKQEENSKKGNRTPFQYL
SpyCas9HNH      DMYVDQELDIN----RLSDYDVDHIVPQSFLKDD-SIDNKVLTRSDKNRGKSDNVPSEEV
AceCas9HNH      CMYCGAPISWE-------NSELDHIVPRTDGGSN-RHENLAITCGACNKEKG-RRPFASW
AnaCas9HNH      CLYCGTTIGYH-------TCQLDHIVPQAGPGSNNRRGNLVAVCERCNRSKS-NTPFAVW
                 :*    :            ::** :* :   .:    * . .    *  *. . *

Nme1Cas9HNH     NGKD--NSREWQEFKARVE--------------TSRFPRSKKQRIL------------
SauCas9HNH      SSSD--SKISYETFKKHILNL---------AKGKGRISKTKKEYLLEE----------
SpyCas9HNH      VKKM--KNYWRQLLNAKLITQRKF--DNLTKAERGGLSELDKAGFIKRQ---------
AceCas9HNH      AETS--NRVQLRDVIDRVQKLKYSGNMYWTRDEFSRYKKSVVARLKRRTSDPEVIQSI
AnaCas9HNH      AQKCGIPHVGVKEAIGRVRGWRKQT-PNTSSEDLTRLKKEVIARLRRTQEDPEIDER-
                  .        .    ::                    .     :
```

RuvC domain

```
CdiCas9RuvC     --------MKYHVGIDVGTFSVGLAAIEVDDAGMPIKTLSLV---SHIHDSGLDPDKIKS
AnaCas9RuvC     -WYASLSAHHLRVGIDVGTHSVGLATLRVDDHGTPIELLSAL---SHIHDSGVGKEGKKD
AceCas9RuvC     ----GTVPVTWRLGVDVGERSIGLAAVSYEED-KPKEILAAV---SWIHDGGVALH----
Nme1Cas9RuvC    MAAFKPNSINYILGLDIGIASVGWAMVEIDEEENPIRLIDLG---VRVFERAEVPKTGKK
SauCas9RuvC     ------MKRNYILGLDIGITSVGYGIIDYETR----DVIDAG---VRLFKEANVEIPTTL
CjeCas9RuvC     --------MARILAFDIGISSIGWAFSENDELKDCGVRIFTK---AENPKTGESLAINED
SpyCas9RuvC     ------MDKKYSIGLDIGTNSVGWAVITDEYKVPSKKFKVLGNTDRHSIKKNLIGALLFD
                            :..*:*  *:* .    :                   .

CdiCas9RuvC     AVTIEPSWTPPAPRIGEPVGNPAVDRVLKTVSRWLESATKTWG--APERVIIEHV-----
AnaCas9RuvC     HDTRKKAIN-------APVGNPSVDRTLKIVGRYLS-AVESWG--TPEVIHVEHVRDGFT
AceCas9RuvC     ----------------EATGHPVVDRNLAILRKFLSSATMRWG--PPQSIVVELY-----
Nme1Cas9RuvC    NTEEKIYLPPIP---ADEIRNPVVLRALSQARKVINGVVRRYG--SPARIHIETAR----
SauCas9RuvC     VD--------------DFILSPVVKRSFIQSIKVINAIIKKYG--LPNDIIIELAR----
CjeCas9RuvC     KKDFLPAFN--ETYYKDEVTNPVVLRAIKEYRKVLNALLKKYG--KVHKINIELAR----
SpyCas9RuvC     SGETAEATDSLHEHIANLAGSPAIKKGILQTVKVVDELVKVMGRHKPENIVIEMAR----
                                     * : : :    : :.      *      : :*

CdiCas9RuvC     SMESVAWMANELRSRVAQHFASH-GTTVRVYRGSLTAEARRASGISGK-----LEFLDGV
AnaCas9RuvC     SSESVAWAN-ELHHRIAAAYP----ETTVVYRGSITAAARKAAGIDSR-----INLIGEK
AceCas9RuvC     -------AAVALRDRLLSYGEKNGVAQVAVFRGGVTAEARRWLDISIERLFSRVAIFAQS
Nme1Cas9RuvC    -------EVGLNDTRYVNRFLCQFVADRMRLTGKGKKRVFASNGQITN----LLRGFWGL
SauCas9RuvC     -------------ERYATRGLMNLLRSYFRVNN-LDVKVKSINGGFTS----FLRRKWKF
CjeCas9RuvC     -YIARLVLNYTKDYLDFLPLSDDENTKLNDTQKGSKVHVEAKSGMLTS----ALRHTWGF
SpyCas9RuvC     ----------RQITKHVAQILDSRMNTKYDENDKLIREVKVITLKSKLVS--DFRKDFQF
                                                      .              .

CdiCas9RuvC     G-KSRLDRRHHAIDAAVIAFTSDYVAETLAVRSNLKQSQAHR-----------QEAPQWR
AnaCas9RuvC     GRKDRIDRRHHAVDASVVALEAS-VAKTLAERSSLRGEQRLT-----------GKEQTWK
AceCas9RuvC     TSTKRLDRRHHAVDAVVLTTLTPGVAKTLADARSRRVSASTE-----------EPQ----
Nme1Cas9RuvC    RKVRAENDRHHALDAVVVACSTVAMQQKITRFVRYKEMNAFDGKTIDKETGEVLHQKTHF
SauCas9RuvC     KKERNKGYKHHAEDALIIANADFIFKEWKKLDKAKKVMENQMFE----------------
CjeCas9RuvC     STKDRNNHLHHAIDAVIIAYANNSIVKAFSDFKKEQESNSAELY----------------
SpyCas9RuvC     YKVREINNYHHAHDAYLNAVVGTALIKKYPKLESEFVYGDYKVYDVRKMIAK-SEQEIGK
                      .  *** ** : :     . :

CdiCas9RuvC     EFTGKDAEHRAAWRVWCQKMEKLSALLTEDLRDDRVVVMSNVR-----------------
AnaCas9RuvC     QYTGSTVGAREHFEWRG--HLHLTELFNERLAEDKVYVTQNIRLRLSD------------
AceCas9RuvC     ---------SPAYRQWKESCSGLGDLLISTAARDSIAVAAPLRLRP--------------
Nme1Cas9RuvC    PQPWEFFAQEVMIRVFGKPDGKPEFEEADTLEKLRTLLAEKLSSRPEAVHEYVTPLFVSR
SauCas9RuvC     -----EKQAESMPEIETEQEYKEIFITPHQIKHIKDFKDYKYSHRV--------------
CjeCas9RuvC     --------AKKISELDYKNKRKFFEPFSGFRQKVLDKIDEIFVSKP--------------
SpyCas9RuvC     ATAKYFFYSNIMNFFKTEITLANGEIRKRPLIETNGETGEIVWDKGRDFATVRKVLSMPQ
```

Supplementary Figure 9

Supplementary Figure 9. Comparison of sequences of the RuvC and HNH domains for the known Cas9s. Sequences are extracted from the domains of the three-dimensional structures of the Cas9. Top, pair-wise sequence identities for the known Cas9. Bottom, sequence alignment for the HNH (top) and the RuvC domain (bottom), respectively. The strictly conserved catalytic residues are highlighted by red outlines.

Table S1a: Statistics of cryo-EM data collection processing

**Data acquisition and processing parameters**	**Data-set 1**	**Data-set 2**
Microscope	Titan Krios G3i	Titan Krios G3i
Detector	Gatan K3	Gatan K3
Voltage	300 kV	300 kV
Collecting mode	Counted super-resolution	Counted super-resolution
Dose rate (e^{-}/ $Å^2$)	60	60
Defocus range (μm)	(-1) - (-2.2)	(-1) - (-2.2)
Nominal magnification	105K	105K
Frames collected per exposure	60	60
Frame-alignment software	MotionCor2	MotionCor2
CTF estimation software	Gctf	Gctf
Raw images collected	5919	7734
Images used for particle picking	5620	7137
2D classification software	Cryosparc	Cryosparc
Final reconstruction software	RELION-4	RELION-4
Applied symmetry	C1	C1
Resolution method	FSC 0.143 cutoff	FSC 0.143 cutoff
Local resolution software	RELION-4	RELION-4
Map visualization software	Pymol/Chimera/Chimera-X, Coot	Pymol/Chimera/Chimera-X, Coot

Table S1b: Statistics of model refinement and data deposition

**Refinement parameters**	**Pre-cleavage**	**Cleavage-intermediate 1**	**Cleavage-intermediate 2**	**Post-cleavage 1**	**Post-cleavage 2**	**Target bound**
Deposited EMDB	**27143**	**27142**	**27141**	**27146**	**27144**	**27145**
CC (mask)	0.71	0.86	0.84	0.79	0.83	0.82
RMSD (Bond lengths/Bond angles)	0.005/0.976	0.005/0.876	0.006/0.907	0.006/0.974	0.005/0.870	0.005/0.805
Number of particles contributed to the final reconstruction	103,063	196,600	186,956	459,996	169,296	91,324
Final resolution (Å)	2.88	2.21	2.43	2.58	2.66	2.78
Ramachandran plot (Outliers<br>Allowed<br>Favored)	0.0<br>4.7<br>95.3	0.0<br>1.8<br>98.2	0.0<br>1.6<br>98.4	0.0<br>3.5<br>96.5	0.0<br>1.6<br>98.4	0.0<br>1.9<br>98.1
Cβ Outliers (%)	0.00	0.00	0.00	0.00	0.00	0.00
ADP (B-factors)<br>Iso/Aniso (#)<br>Protein<br>Nucleotide<br>Ligand<br>Water<br>(min/mask/mean)	11415/0<br>0.0/52.7/19.2<br>0.0/68.2/24.2<br>17.8/17.8/17.8<br>---	12228/0<br>0.0/61.9/24.6<br>0.0/119.0/40.3<br>14.5/63.0/47.1<br>2.2/30.81/18.1	12295/0<br>0.0/63.7/27.8<br>0.0/127.2/44.7<br>13.8/79.4/60.7<br>2.6/48.6/20.2	9722/0<br>0.00103.2/38.2<br>1.7/186.4/70.6<br>15.8/150.9/69.6<br>24.4/32.6/28.5	8472/0<br>0.0/71.2/34.1<br>6.1/123.1/57.1<br>24.4/56.3/34.5<br>21.6/41.8/33.8	11436/0<br>0.0/59.4/25.2<br>0.0/116.8/37.1<br>6.6/69.0/54.2<br>0.0/29.3/14.7
MolProbity score	1.61	1.15	1.18	1.50	1.17	1.16
Clash score	5.26	3.65	3.93	5.23	3.83	3.38
Rotamer outlier (%)	0.00	0.00	0.00	0.00	0.00	0.00
FSC model (0/0.143/0.5)	2.4/2.5/3.1	2.0/2.0/2.3	2.0/2.1/2.5	2.0/2.2/2.7	2.2/2.3/2.8	2.2/2.3/2.8
Deposited PDB codes	**8D2N**	**8D2L**	**8D2K**	**8D2Q**	**8D2O**	**8D2P**

Supplementary Table 2. DNA or RNA Oligos used for this study

Name	Sequence (5'-3')	Used for
**40mer NTS**<br>**40mer TS**	TCTAGAGGTAGGATGGCAAGATCCTGGTATACACCAAGCT<br>AGCTTGGTGTATACCAGGATCTTGCCATCCTACCTCTAGA	Cryo EM
**DNA for sgRNA106**	GAA CCC CCT CGC TGC TGC GAG GGGGTG AAG AAT GCG ACC CCA CGA AGGGGT CTT GCT AGG TAG CCT TTT CAGGCT CCC CAG CAT ACC AGG ATC TTGCCA TCC TAC CTA TAG TGA GTC GTATTA	Cryo EM
**sgRNA106**	GGUAGGAUGGCAAGAUCCUGGUAUGCUGGGGAGCCUGAAAAGGCUACCUAGCAAGACCCCUUCGUGGGGUCGCAUUCUUCACCCCCUCGCAGCAGCGAGGGGGUUC	Cryo EM
**FAM NTS**<br>**TS**	[FAM] GGT AGG ATG GCA AGA TCC TGGTAT ACA CCA AGC T<br>AGC TTG GTG TAT ACC AGG ATC TTGCCA TCC TAC C	Oligo Cleavage assay
**NTS**<br>**HEX TS**	TCTAGAGGTAGGATGGCAAGATCCTGGTATACACCAAGCT<br>[HEX] AGCTTGGTGTATACCAGGATCTTGCCATCCTACCTCTAGA	Oligo Cleavage assay
**G685E-F**<br>**G685E-R**	GAATCCACCGAATACGCAGCTG<br>AATGCTTTGAATCACTTCGGG	Q5 site-directed mutagenesis
**V709E-F**<br>**V709E-R**	CAGGTAGCGGAATTCCGCGGTG<br>AGCTACTCCGTTCTTTTCGCCG	Q5 site-directed mutagenesis
**D674Y-F**<br>**D674R-F**<br>**D674Y_R-R**	CGCACCTCCTATCCCGAAGTG<br>CGCACCTCCCGTCCCGAAGTG<br>CCTTTTGAGGCGGGCGACGAC	Q5 site-directed mutagenesis
**D770R-F**<br>**D770R-R**	GAAGACCTTGGCCAGAGCGCGGAGTC<br>GCGACGCCCGGAGTCA	Q5 site-directed mutagenesis
**R295A-F**<br>**R295A-R**	GCTAACCTGGCGATACGTGATGG<br>CACCGCCGCAACGATGCGATAC	Q5 site-directed mutagenesis
**R744E_F**<br>**R744E_R**	GCACGAAGGAACTCGATCGTCGGC<br>TCGTTGACTGAGCAAAAATTGC	Q5 site-directed mutagenesis
**R619E_F**<br>**R619E_R**	GGA AAA AGG TGA ACG TCC CTT TGCG<br>TTG TTG CAC GCT CCG CAG G	Q5 site-directed mutagenesis
**H750N_F**<br>**H750n_R**	CGT CGG CAC AAC GCC GTG GAC GCG<br>ATC GAG CCG CTT CGT GCT CGT TGA C	Q5 site-directed mutagenesis
**H750D_F**<br>**H750D_R**	GTC GGC ACG ATG CCG TGG ACG CG<br>GAT CGA GCC GCT TCG TGC TCG TTG	Q5 site-directed mutagenesis
